# Cycle threshold values and SARS-CoV-2 variant associations with breakthrough infections: a retrospective study in Accra, Ghana

**DOI:** 10.1186/s12879-025-11732-6

**Published:** 2025-10-10

**Authors:** Frank Twum Aboagye, Lawrence Annison, Ebenezer Krampah Aidoo, Maame Ekua Acquah, Yvonne Aryeetey Ashong, Betty Bandoh Oppong, Lawrencia Osae-Nyarko, Isaac Owusu-Frimpong, Henry Kwadwo Hackman, Sharon Annison, Queenstar Dedei Quarshie, Abena Konadu Owusu-Senyah Enninful, Naa Adjeley Kuma, Bill Clinton Egyam, Mike Y. Osei-Atweneboana

**Affiliations:** 1 Department of Medical Laboratory Technology, Faculty of Applied Sciences, Accra Technical University, Accra, Ghana; 2 Biomedical and Public Health Research Unit, Council for Scientific and Industrial Research – Water Research Institute, Accra, Ghana; 3 West African Centre for Cell Biology of Infectious Pathogens, College of Basic and Applied Sciences, University of Ghana, Legon, Accra Ghana; 4 Department of Parasitology, College of Medical Sciences, Noguchi Memorial Institute of Medical Research, University of Ghana, Legon, Accra Ghana; 5 Department of Molecular Microbiology and Immunology, Bloomberg School of Public Health, Johns Hopkins University, Baltimore, MD United States of America; 6 Department of Epidemiology and Disease Control, School of Public Health, University of Ghana, Legon, Accra Ghana; 7 Department of Molecular Biology, MDS Lancet Laboratories Ghana Limited, East Legon, Accra, Ghana; 8 Council for Scientific and Industrial Research – College of Science and Technology, Accra, Ghana

**Keywords:** SARS-CoV-2, Breakthrough infection, Ghana, COVID-19 vaccination, Variants

## Abstract

**Background:**

Breakthrough infections are defined as SARS-CoV-2 infections occurring ≥ 14 days after completing the primary COVID-19 vaccination series and remain a public health challenge, particularly in regions where immune-evasive variants are circulating. However, data on their virological and clinical profiles in low-resource settings are limited.

**Methods:**

This retrospective study was conducted from July to December 2022 in Accra, Ghana, among individuals testing positive for SARS-CoV-2. Real-time Reverse Transcription Polymerase Chain Reaction (RT-PCR) was performed using the Allplex™ 2019-nCoV Assay. Cycle threshold (Ct) values for the nucleocapsid (N), RNA-dependent RNA polymerase (RdRP), and envelope (E) genes, categorised as < 25, 25–30, or > 30. Variant identification targeted Alpha, Delta, and Omicron mutations using mutation-specific RT-PCR. Logistic regression was used to assess associations between vaccination status and demographic, clinical, and virological factors.

**Results:**

Of the 268 samples analysed, 81 tested positive; 43.20% [*n* = 35] were vaccinated individuals. Median Ct-values for the N [27.13, IQR: 21.59–31.96] and E [24.57, IQR: 19.43–29.43] genes were significantly higher among vaccinated cases, indicating lower viral loads. Breakthrough infections were strongly associated with the Omicron variant [aOR = 4.38, *p* = 0.034]. Diarrhoea [aOR = 9.67, *p* = 0.022], sore throat [aOR = 8.99, *p* = 0.038], headache [aOR = 10.156, *p* = 0.039] and chills [aOR = 3.316, *p* = 0.046] were mostly associated with breakthrough infections. Ct-values of 25–30 [aOR = 11.33, *p* = 0.012] and > 30 [aOR = 4.01, *p* = 0.047] were significantly associated with breakthrough infection compared to Ct < 25 in breakthrough infections.

**Conclusion:**

Vaccinated individuals with SARS-CoV-2 infection had lower viral loads and were more likely to be infected with the Omicron variant. These findings reinforce the role of vaccination in reducing viral load and support the adoption of practical surveillance strategies, such as Ct value-based surveillance and variant screening in low middle-income countries facing similar constraints in genomic capacity and vaccine deployment.

**Supplementary Information:**

The online version contains supplementary material available at 10.1186/s12879-025-11732-6.

## Introduction

Breakthrough infections, defined as SARS-CoV-2 infections occurring in fully vaccinated individuals [1], have emerged as a significant concern during and after the COVID-19 pandemic. Although vaccination remains effective in preventing severe illness and death, the occurrence of post-vaccination infections, particularly in the presence of variants of concern (VOCs), poses challenges to sustained transmission control [2]. Variants such as Delta and Omicron have demonstrated increased transmissibility and partial escape from vaccine-induced neutralising antibodies, thereby undermining vaccine effectiveness against infection [3–5].

Viral load, a key determinant of transmissibility, is typically inferred from cycle threshold (Ct) values obtained through real-time RT-PCR assays. Lower Ct values reflect higher viral loads and greater potential for transmission, even in vaccinated individuals [6, 7]. Several studies have shown that vaccinated individuals who experience breakthrough infections tend to exhibit higher Ct values, suggesting lower viral burden and possibly reduced infectiousness [8–10]. However, the generalisability of these findings remains limited by the fact that most of the existing evidence originates from high-income countries, where mRNA-based vaccines dominate, and healthcare systems are better resourced.

In contrast, low- and middle-income countries (LMICs), including those in sub-Saharan Africa, face contextual challenges that may alter the epidemiological and virological landscape of breakthrough infections. These include limited vaccine access, delayed booster rollout, reliance on viral vector vaccines, and underdeveloped genomic surveillance infrastructure. Recent analyses of SARS-CoV-2 evolution have also shown that viral diversification and clustering patterns differ across regions, including Africa, which may influence the circulation of variants and the risk of breakthrough infection [11]. These disparities highlight the need for region-specific data to inform public health strategies that are responsive to local realities.

In Ghana, recent molecular surveillance from the post-lockdown period (July to December 2022) in the Accra Metropolis reported a SARS-CoV-2 positivity rate of 30.2%, with the Alpha variant being the most prevalent, followed by Delta and Omicron [12]. While Alpha dominated locally, global trends during the same period showed rapid expansion of immune-evasive variants such as Omicron [11], illustrating how regional evolutionary trajectories may diverge from global patterns. Higher infection rates were observed among females and adults aged 41–50 years, while lower Ct values, indicative of high viral load, were reported in children under 10 years. Despite these findings, limited data exist on how Ct values, variant type, and clinical presentation differ between vaccinated and unvaccinated individuals in Ghana.

Given this gap, the present study aimed to characterise the virological and clinical features of SARS-CoV-2 breakthrough infections in a Ghanaian cohort. Specifically, we (1) compared cycle threshold (Ct) values between vaccinated and unvaccinated individuals; (2) evaluated the distribution of SARS-CoV-2 variants among breakthrough cases; and (3) examined clinical predictors, including symptom profiles, associated with breakthrough infection. We hypothesised that breakthrough infections would be associated with higher Ct values (indicating lower viral loads) and a higher prevalence of immune-evasive variants such as Omicron.

This study provides novel data that not only inform public health planning in Ghana but are also broadly applicable to other low- and middle-income countries (LMICs) that face similar constraints in vaccine access, genomic surveillance, and healthcare infrastructure. In particular, the findings have relevance for LMICs where vector-based vaccines are predominant and booster rollout remains limited.

## Methods

### Study design and setting

This was a retrospective study involving secondary analysis of laboratory and clinical data collected during a previous study conducted between July and December 2022 at MDS Lancet Laboratories Limited, a private ISO-certified diagnostic facility in Accra, Ghana. The laboratory serves as a major COVID-19 testing centre in the region and routinely conducts molecular testing and variant screening for SARS-CoV-2. The study targeted individuals who tested positive for SARS-CoV-2 by real-time RT-PCR during the specified period. Participants were selected from a larger molecular epidemiology cohort described previously by Aboagye et al. [12]. Inclusion was limited to individuals with complete COVID-19 vaccination records to allow for accurate classification of breakthrough infections and analysis of virological characteristics, including cycle threshold (Ct) values and variant distribution.

### Study population

The study population comprised individuals who tested positive for SARS-CoV-2 by real-time reverse transcription polymerase chain reaction (RT-PCR) at MDS Lancet Laboratories between July and December 2022. Eligibility criteria included (1) a confirmed positive RT-PCR result for SARS-CoV-2, and (2) availability of complete COVID-19 vaccination records, including dates of vaccine administration. Breakthrough infections were defined according to the Centre for Disease Control and Prevention criteria as RT-PCR-confirmed SARS-CoV-2 infections occurring ≥ 14 days after receipt of all recommended doses of a COVID-19 vaccine [1]. Individuals who lacked verifiable vaccination information or tested positive within 14 days of their last vaccine dose were excluded. This selection allowed for a focused evaluation of virological characteristics, specifically Ct values and variant type among fully vaccinated individuals in comparison with unvaccinated cases.

Participants were categorised into age groups for analysis. The ≤ 20 years group [*n* = 12] included children and adolescents aged 5–20 years, while the > 60 years group [*n* = 9] comprised older adults. No infants were included in the study. Given the small number of participants in these categories, findings for the age groups should be interpreted with caution, and generalisations are limited.

### Data collection

Data were retrospectively extracted from the clinical and laboratory data, previously compiled as part of a molecular epidemiology study conducted by Aboagye et al., (2024). The dataset included demographic variables (age and sex), vaccination history, vaccine type clinical presentation (symptomatic vs. asymptomatic), and the presence of individual symptoms as reported at the time of testing. The vaccines administered in the study population were Johnson & Johnson (single-dose viral vector), AstraZeneca (two-dose viral vector), Sputnik V (two-dose viral vector), and Pfizer-BioNTech (two-dose mRNA).

Laboratory variables included cycle threshold (Ct) values for three SARS-CoV-2 gene targets: nucleocapsid (N), RNA-dependent RNA polymerase (RdRP), and envelope (E). Ct values were used as a proxy for viral load and categorised into three groups based on established thresholds: <25 (high viral load/severe), 25–30 (moderate viral load), and >30 (low viral load), consistent with previously published classifications [13, 14]. Ct values were derived from diagnostic RT-PCR tests performed at the first point of detection for each participant. Only the initial sample collected at diagnosis was included in the analysis, ensuring consistency in the timing of Ct measurement across individuals. For symptomatic participants, this generally corresponded to the time of presentation or testing for COVID-19, while asymptomatic individuals were identified through contact tracing and routine screening. No follow-up Ct values were used in this analysis. Symptomatic status at the time of testing was also recorded and incorporated into subgroup analyses.

**Fig. 1:**
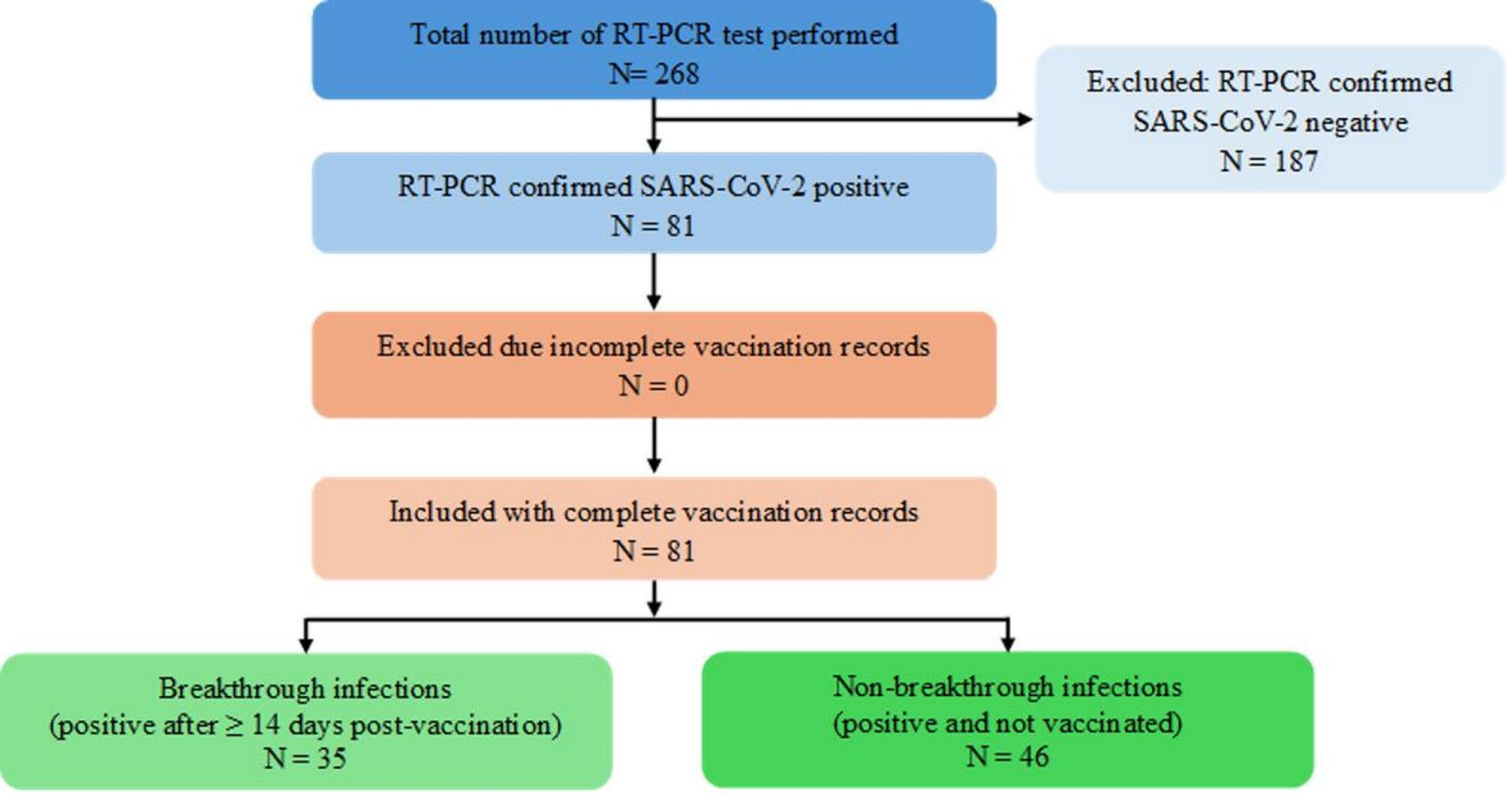
Flowchart of data collection and study population selection. The chart outlines the total RT-PCR tests performed, SARS-CoV-2 positive cases, and classification based on vaccination status

Variant identification data, classifying samples as Alpha, Delta, or Omicron were also extracted for participants who underwent mutation-specific PCR typing. All personal identifiers were removed during data extraction, and anonymised datasets were used for analysis. A schematic overview of the participant selection process, including RT-PCR testing outcomes and classification by vaccination status, is presented in Fig. 1.

### Sample collection

Nasopharyngeal specimens were collected by trained personnel using sterile nylon-tipped flocked swabs, following standard clinical protocols for upper respiratory sampling [15]. The swab was inserted into the participant’s nostril and advanced to the posterior nasopharynx, where it was gently rotated to absorb epithelial cells and viral particles.

Collected specimens were immediately placed in viral transport medium (VTM) (Shanghai Focusgen Biotechnology Co., Ltd China), then stored at 2–8 °C and transported to the COVID-19 testing facility of MDS Lancet Laboratories under cold-chain conditions. All procedures followed international guidelines for respiratory virus sample collection, handling, and biosafety [15, 16].

### Laboratory analysis

Detection of SARS-CoV-2 RNA was performed using real-time reverse transcription polymerase chain reaction (RT-PCR) assays targeting three genomic regions: nucleocapsid (N), RNA-dependent RNA polymerase (RdRP), and envelope (E) genes. Viral RNA was extracted using the Zymo Quick-RNA™ Viral Kit (Zymo Research Corporation, USA) according to the manufacturer’s instructions with minor protocol modifications as previously described [17]. Amplification was conducted using the Allplex™ 2019-nCoV Assay (Seegene Inc., South Korea) on a CFX96 Real-Time PCR System (Bio-Rad, USA). Synthetic RNA controls were included in every run, and results were accepted only when Ct values remained within ± 1 cycle of the expected range. This ensured inter-run standardisation and reliability of Ct value comparisons across samples.

The Allplex™ 2019-nCoV (Seegene Inc., South Korea) assay reports a clinical sensitivity of 97.1% and specificity of 100% based on internal validation and external comparative studies [18]. The assay includes built-in positive, negative, and internal controls to ensure run validity and detect PCR inhibition.

To enable semi-quantitative estimation of viral load, each RT-PCR run included a serial dilution of synthetic SARS-CoV-2 RNA standards with known copy numbers. Ct values for each target gene were interpolated against a standard curve to estimate viral RNA copy numbers per millilitre. For comparative purposes, Ct values were categorised as follows: Ct < 25 (high viral load), Ct 25–30 (moderate viral load), and Ct >30 (low viral load), based on previously established cutoffs [13, 14].

Variant identification was performed using mutation-specific RT-PCR assays targeting key single nucleotide polymorphisms (SNPs) characteristic of WHO-designated variants of concern. These included N501Y and Δ69–70 (Alpha), L452R and P681R (Delta), and multiple spike protein mutations for Omicron [19]. This approach has demonstrated over 95–100% concordance with whole-genome sequencing in prior comparative evaluations [20, 21]. Typing was successfully performed for all 81 participants included in the study, ensuring a complete dataset for correlating variant type with clinical features and Ct values. All laboratory analyses were conducted in accordance with international biosafety standards and quality assurance protocols.

### Data analysis

Statistical analysis was conducted using SPSS version 27 (IBM Corp., Armonk, NY, USA) and GraphPad Prism version 8.0 (GraphPad Software, San Diego, CA, USA). Only participants with complete clinical, demographic, Ct value, and variant data were included in the analysis. No imputation was required or applied, and no data were excluded due to incompleteness. Continuous variables, including Ct values and age, were assessed for distribution and summarised as medians with interquartile ranges (IQRs). Comparisons between vaccinated and unvaccinated groups were performed using the Mann–Whitney U test for non-parametric data. Categorical variables, such as sex, clinical presentation, and variant type, were summarised as frequencies and percentages and compared using the chi-square test or Fisher’s exact test where appropriate.

Vaccines were classified as either viral vector vaccine (Johnson and Johnson, Sputnik and AstraZeneca) or mRNA (Moderna and Pfizer-B) vaccine depending on the brand of vaccine the participant had taken at the time of the study. To evaluate the impact of vaccination timing on breakthrough infection, the time interval between the last vaccine dose and SARS-CoV-2 diagnosis was categorised into three groups: 14–30 days, 31–60 days, and >60 days. This classification reflects early, intermediate, and waning periods of vaccine-induced immunity and was informed by published studies on post-vaccination viral kinetics [2, 9].

A binary logistic regression model was used to identify factors independently associated with breakthrough infection, defined as SARS-CoV-2 infection occurring ≥ 14 days after completion of the primary COVID-19 vaccination series [1]. Explanatory variables included SARS-CoV-2 variant type, Ct value category, symptom status (symptomatic vs. asymptomatic), and individual symptoms. Variables were selected based on biological plausibility and prior evidence of association with breakthrough risk. Multicollinearity was assessed using Variance Inflation Factor (VIF); all variables had VIF values below 2, indicating no significant collinearity. Adjusted odds ratios (aORs) with corresponding 95% confidence intervals (CIs) were reported to measure the strength of associations. A p-value of < 0.05 (two-sided) was considered statistically significant.

## Results

### Participant characteristics

A total of 81 individuals were included in the analysis. Of these, 46 [56.79%] were female and 35 [43.21%] were male (Table 1). The median age of participants was 40 years [IQR: 28.50–50.50]. The largest age group was 21–40 years, representing 39.51% of the sample, followed by individuals aged 41–60 years [34.57%]. Participants aged ≤ 20 years accounted for 14.81%, while those over 60 years constituted 11.11% (Table 1). At the time of diagnosis, 43 participants [53.09%] were symptomatic, while 38 [46.91%] were asymptomatic. Among symptomatic individuals, the most frequently reported symptom was cough [43.21%], followed by sore throat [27.16%], headache [25.93%], and chills [19.75%] (Table 1). Other symptoms reported included runny nose [13.58%], fever [8.64%], muscle pain [8.64%], body weakness [4.94%], diarrhoea [3.70%], and joint pain [2.47%]. Symptoms reported were not mutually exclusive as some participants reported more than one symptom simultaneously as shown in Table 1.

**Table 1:** Demographic and clinical characteristics of study participants

Variables	Frequency	Percentage
Gender		
Male	35	43.21
Female	46	56.79
Age (years)		
Median age (years)	40 [IQR: 28.50–50.00]	
≤ 20 years	12	14.81
21–40	32	39.51
41–60	28	34.57
> 60 years	9	11.11
Clinical Presentation		
Symptomatic	43	53.09
Asymptomatic	38	46.91
Symptoms Presented*		
Chills	16	19.75
Cough	35	43.21
Diarrhoea	3	3.70
Fever	7	8.64
Sore Throat	22	27.16
Headache	21	25.93
Joint pain	2	2.47
Muscle pain	7	8.64
Runny nose	11	13.58
Body weakness	4	4.94

*Symptoms were not mutually exclusive; some participants reported more than one symptom

### Prevalence and distribution of breakthrough infections

Demographic and clinical characteristics of SARS-CoV-2–infected individuals were analysed by vaccination status (Table 2). Vaccination characteristics, including vaccine brand, dose, and interval from last vaccination to sample collection, are summarised in Table S1. Among the 81 participants, 43.2% [*n* = 35] were vaccinated and 56.8% [*n* = 46] were unvaccinated (Table 2). Vaccination rates were comparable between males [45.7%] and females [41.3%], with no statistically significant difference by sex [χ^2^ = 2.782, *p* = 0.095] (Table 2). Age-stratified analysis showed that unvaccinated individuals were more in the younger (≤ 20 years) and middle-aged (41–60 years) groups. In the ≤ 20 age group, 75.0% of infections occurred in unvaccinated individuals [ꭓ^2^ = 6.000, *p* = 0.014], while in the 41–60 age group, 64.3% were unvaccinated [χ^2^ = 4.571, *p* = 0.033]. No significant difference in vaccination status was observed in participants over 60 years [χ^2^ = 1.890, *p* = 0.169] (Table 2). Although these age-related trends suggest differences in vaccine uptake or risk exposure, the small subgroup sizes warrant cautious interpretation.

Symptomatic infection was significantly more common among unvaccinated individuals [67.4%] compared to those vaccinated [32.6%; χ^2^ = 10.465, *p* = 0.001] (Table 2). Among reported symptoms, cough, headache, and runny nose were notably more frequent in the unvaccinated group (Table 2). Cough was observed in 68.6% of unvaccinated cases versus 31.4% of vaccinated cases [χ^2^ = 9.657, *p* = 0.002], headache in 71.4% versus 28.6% [χ^2^ = 7.71-4, *p* = 0.006], and runny nose in 81.8% versus 18.2% [χ^2^ = 8.909, *p* = 0.003], respectively (Table 2). Details of the prevalence and distribution of SARS-CoV-2 infection between vaccinated and unvaccinated participants is presented in Table 2.

**Table 2:** Distribution of demographic and clinical features among vaccinated and unvaccinated SARS-CoV-2–infected individuals in Accra

Variable	Total	Vaccinated		Unvaccinated		ꭓ^2^ [*p*-values]
		*n*	% [95% CI]	*N*	% [95% CI]	
Gender						0.158 [0.691]
Male	35	16	45.71 [30.41–61.90]	19	54.29 [38.09–69.59]	
Female	46	19	41.30 [28.25–55.73]	27	58.70 [44.26–71.74]	
Age (years)						4.882 [0.181]
≤ 20	12	3	25.00 [18.89–53.23]	9	75.00 [46.77–91.11]	
21–40	32	16	50.00 [33.63–66.37]	16	50.00 [33.63–66.37]	
41–60	28	10	35.71 [20.71–54.17]	18	64.29 [45.83–79.29]	
> 60	9	6	66.67 [35.42–87.94]	3	33.33 [12.06–64.58]	
Clinical Presentation						**4.238 [0.040]**
Symptomatic	43	14	32.56 [20.49–47.48]	29	67.44 [52.52–79.51]	
Asymptomatic	38	21	55.26 [39.71–69.85]	17	44.74 [30.15–60.29]	
Symptoms Presented*						
Chills	16	6	37.50 [18.48–61.36]	10	62.50 [38.64–81.52]	1.937 [0.164]
Cough	35	11	31.43 [18.55–47.98]	24	68.57 [52.02–81.45]	**9.657 [0.002]**
Diarrhoea	12	7	58.33 [31.95–80.67]	5	41.67 [19.33–68.05]	0.667 [0.414]
Fever	19	7	36.84 [19.15–58.96]	12	63.16 [41.04–80.85]	2.632 [0.105]
Sore Throat	22	9	40.91 [23.26–61.27]	13	59.09 [38.73–76.74]	1.455 [0.228]
Headache	21	6	28.57 [13.81–49.96]	15	71.43 [50.04–86.19]	**7.714 [0.006]**
Joint pain	13	5	38.46 [17.71–64.48]	8	61.54 [35.52–82.29]	1.385 [0.239]
Muscle pain	7	3	42.86 [15.82–74.95]	4	57.14 [25.05–84.18]	0.286 [0.593]
Runny nose	11	2	18.18 [15.14–47.70]	9	81.82 [52.30–94.86]	**8.909 [0.003]**
Body weakness	25	12	48.00 [30.03–66.50]	13	52.00 [33.50–69.97]	0.080 [0.777]

Boldened values are significant at p < 0.05

*95% CI* 95% Confidence Interval, *ꭓ*^2^ Chi square

* Multiple responses were submitted by each participant on the symptoms they experienced

### Association of breakthrough infection with disease severity (Ct-value)

**Fig. 2:**
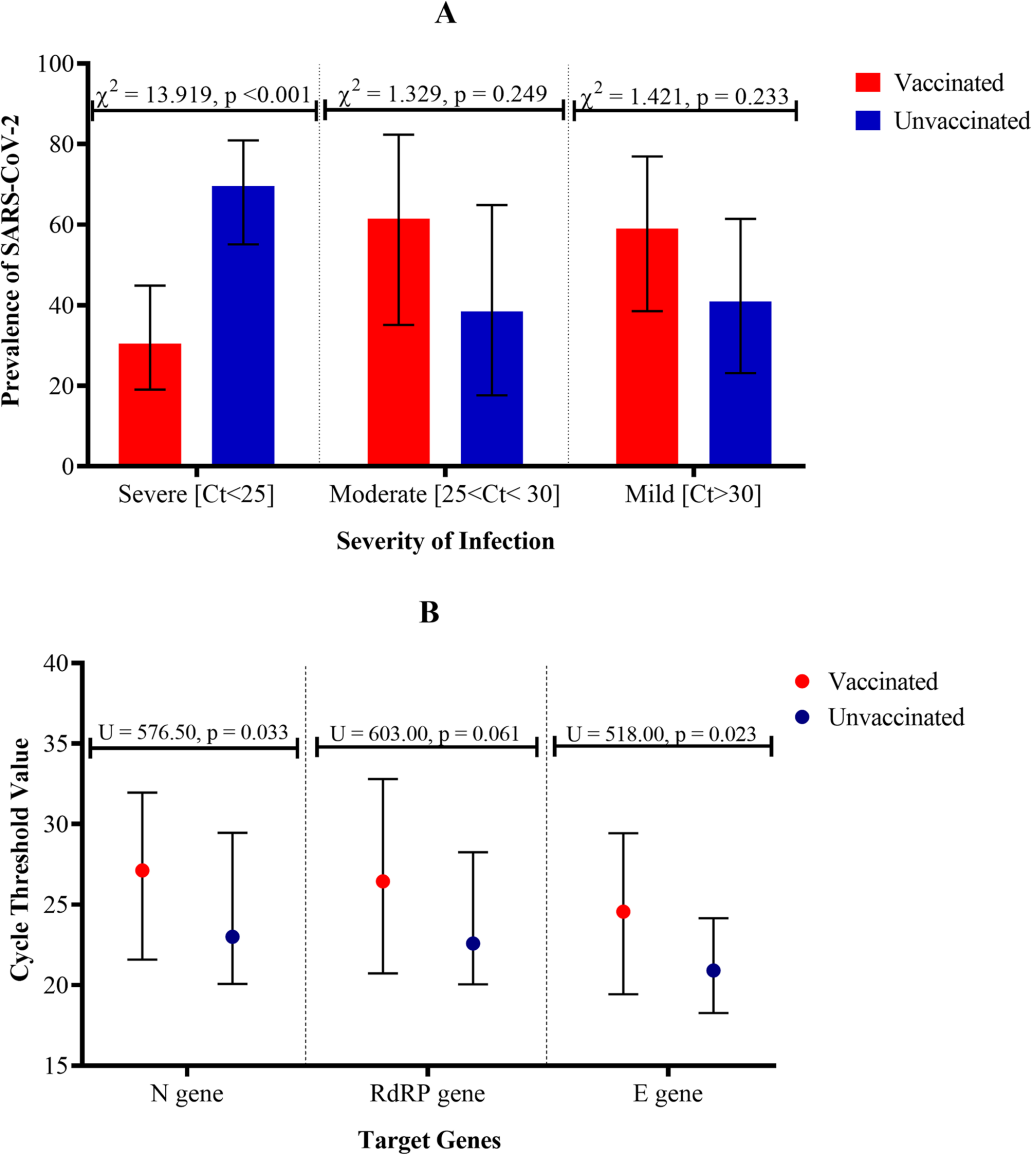
SARS-CoV-2 infection characteristics among vaccinated (red) and unvaccinated individuals (blue). **A** Distribution of infection severity based on cycle threshold (Ct) categories: severe (Ct < 25), moderate (25 ≤ Ct < 30), and mild (Ct ≥ 30). Prevalence is compared between vaccinated (breakthrough infections) and unvaccinated individuals using Chi-square test. **B** Median Ct values and interquartile ranges for N, RdRP, and E genes, comparing vaccinated (red) and unvaccinated (blue) individuals. Mann–Whitney U test was used to assess statistical differences

Figure 2 presents the distribution of SARS-CoV-2 infection characteristics among vaccinated and unvaccinated individuals with a focus on the severity of the infection. The prevalence of SARS-CoV-2 infection with Ct values < 25 (indicative of high viral load) was significantly [ꭓ^2^ = 13.919, *p* < 0.001] lower among vaccinated individuals [30.43%, 95% CI: 19.08–44.89] compared to unvaccinated individuals [69.54%, 95% CI: 55.10–80.91] as shown in Fig. 2A. However, in SARS-CoV-2 infections with Ct values between 25 and 30 (indicative of moderate viral load), the prevalence was higher in vaccinated participants [61.53%, 95% CI: 35.14–82.34] relative to unvaccinated participants [38.47%, 95% CI: 17.66–64.86]. Notwithstanding this, the difference was not statistically significant [ꭓ^2^ = 1.329, *p* = 0.249] (Fig. 2A). Similarly, in SARS-CoV-2 infections with Ct-values > 30 (indicative of low viral load), the prevalence in vaccinated participants [59.09%, 95% CI: 38.54–76.81] was not significantly [ꭓ^2^ = 1.421, *p* = 0.233] higher as compared to unvaccinated participants [40.91%, 95% CI: 23.19–61.46] as shown in Fig. 2A.

Furthermore, Fig. 2B presents that the median Ct values for the N gene was significantly [U = 576.50, *p* = 0.033] higher in vaccinated participants [27.13, IQR: 21.59–31.96] compared to unvaccinated participants [23.00, IQR: 20.08–29.46]. Similarly, the median Ct-value of RdRP gene reported for vaccinated participants [26.45, IQR: 20.73–32.80] was higher than that reported for unvaccinated participants [22.59, IQR: 20.05–28.26]. The difference reported was not statistically significant [U = 603.00, *p* = 0.061]. More so, E gene were significantly [U = 518.00, *p* = 0.023] higher in the vaccinated participants [24.57, IQR: 19.43–29.43] as compared unvaccinated participants [20.91, IQR: 18.27–24.16] as shown in Fig. 2B.

(Ct) categories: severe (Ct < 25), moderate (25 ≤ Ct < 30), and mild (Ct ≥ 30).(Ct) categories: severe (Ct < 25), moderate (25 ≤ Ct < 30), and mild (Ct ≥ 30).

**Fig. 3:**
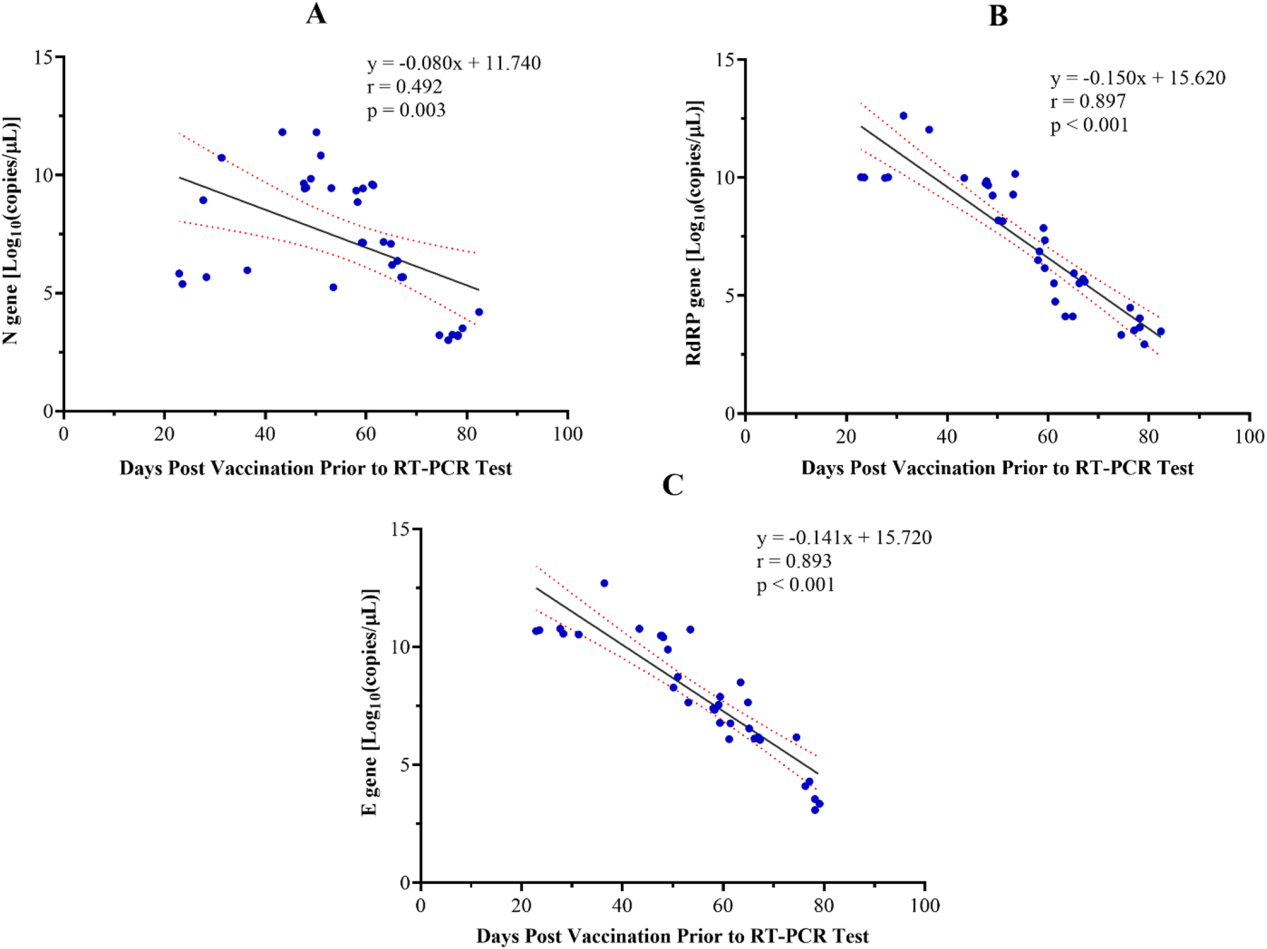
Relationship between days post-vaccination and SARS-CoV-2 viral load (log₁₀ copies/mL) across three target genes: N gene (**A**), RdRP gene (**B**), and E gene (**C**)

The relationship between days post-vaccination prior to RT-PCR positive test and SARS-CoV-2 viral load across the target genes is presented in Fig. 3. A negative linear correlation was observed between the number of days post-vaccination and the estimated SARS-CoV-2 viral load (log_10_copies/mL) for all three target genes. As shown in Fig. 3A, a moderately significant association was observed between days since vaccination and the estimated viral load for the N gene [*r* = − 0.492, *p* = 0.003]. However, a stronger and statistically significant inverse relationship was observed for the RdRP gene [*r* = − 0.897, *p* < 0.001] as shown in Fig. 3B. A similar pattern was observed for the E gene (Fig. 3C), where the association was significantly strong [*r* = − 0.93, *p* < 0.001].

**Fig. 4:**
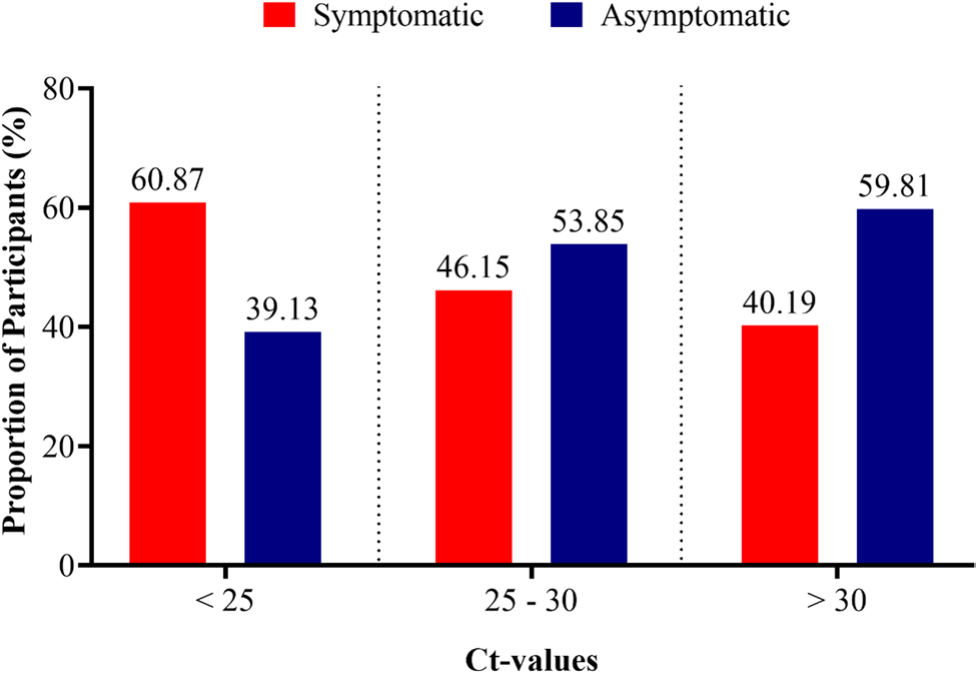
Distribution of cycle threshold values (Ct-values) by symptoms status

To explore whether symptom status was associated with Ct values, participants were stratified into symptomatic and asymptomatic groups across Ct categories (< 25, 25–30, > 30). As shown in Fig. 4, 60.87% of participants with Ct values < 25 were symptomatic compared with 39.13% who were asymptomatic. In the 25–30 category, the proportions were more evenly distributed [46.15% symptomatic vs. 53.85% asymptomatic], while in the > 30 category, a greater proportion were asymptomatic [59.81%] compared with symptomatic [40.19%]. Although symptomatic participants appeared more frequent at lower Ct values, a chi-square test of independence showed that the differences in symptom distribution across Ct categories were not statistically significant [χ^2^ = 2.680, *p* = 0.262].

#### Age-Adjusted Ct-Value analysis and association with breakthrough infection

To explore the potential confounding effect of age on viral load, Ct values were stratified by age group and vaccination status (Table 3). Among the ≤ 21-year-old, the Ct-values were evenly distributed among the unvaccinated participants, whereas vaccinated participants predominantly had Ct values < 25 [2/3, 66.67%]. As shown in Table 3, 56.25% [*n* = 9] of the vaccinated participants aged 21–40 years had Ct-values above 30, while 75.00% [*n* = 12] of the unvaccinated participants aged 21–40 years had Ct-values < 25 (Table 3). In the 41–60 and > 60 years groups, unvaccinated participants generally exhibited lower Ct values, indicative of higher viral loads, compared with vaccinated participants (Table 3).

**Table 3:** Distribution of Ct values by age group and vaccination status among SARS-CoV-2-infected individuals

Age group (years)	Vaccination status	N	Cycle threshold (Ct-value) n [%]		
			< 25	25–30	> 30
≤ 20	Unvaccinated	9	3 [33.33]	3 [33.33]	3 [33.33]
	Vaccinated	3	2 [66.67]	1 [33.33]	0 [00.00]
21–40	Unvaccinated	16	12 [75.00]	0 [00.00]	4 [25.00]
	Vaccinated	16	4 [25.00]	3 [18.75]	9 [56.25]
41–60	Unvaccinated	18	14 [77.78]	2 [11.11]	2 [11.11]
	Vaccinated	10	6 [60.00]	2 [20.00]	2 [20.00]
> 60	Unvaccinated	3	3 [100.00]	0 [00.00]	0 [00.00]
	Vaccinated	6	2 [33.33]	2 [33.33]	2 [33.33]
Overall	Unvaccinated	46	32 [69.57]	5 [10.87]	9 [19.56]
	Vaccinated	35	14 [40.00]	8 [22.90]	13 [37.10]

*N* total number examined, *n* number of positives, *%* percentage

To formally account for potential confounding by age in the relationship between vaccination status and viral load, a linear regression was performed with N gene Ct value as the dependent variable, vaccination status as the independent variable, and age as a covariate (Table 4). Vaccinated participants had significantly higher Ct values compared to unvaccinated participants [β = 3.11; 95% CI: 0.48–5.73; *p* = 0.021], while age was not significantly associated with Ct values [β = −0.72; 95% CI: −2.21–0.77; *p* = 0.338] (Table 4). These findings indicate that the observed differences in viral load were primarily attributable to vaccination rather than age among the participants.

**Table 4:** Age-adjusted linear regression of Ct values and vaccination status

Variable	β	SE	t	*p*-value	95% CI
Constant	25.903	1.943	13.333	< 0.001	22.035–29.771
Vaccination Status	3.106	1.319	2.354	0.021	0.479–5.733
Age (years)	−0.721	0.749	−0.963	0.338	−2.212–0.769

*β* Coefficient of regression, *SE* Standard Error, *95% CI* 95% Confidence Interval

### Variant distribution and breakthrough infection

**Fig. 5:**
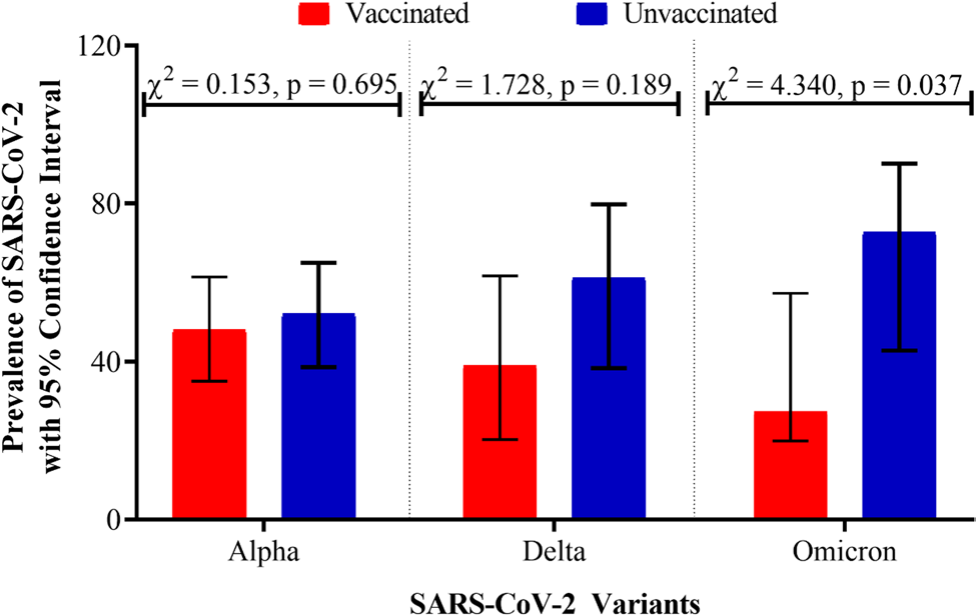
Distribution of SARS-CoV-2 variants among vaccinated and unvaccinated participants

Three SARS-CoV-2 variants were identified in the parent study (Aboagye et al., 2024), thus Alpha [64.2%], Delta [22.22%] and Omicron [13.6%]. This study further explored the distribution of the SARS-CoV-2 among breakthrough cases and non-breakthrough cases (unvaccinated). As shown in Fig. 5, the prevalence of the Alpha variant among the breakthrough cases was 48.07% [95% CI: 35.06–61.36] and 51.93% [95% CI: 38.63–64.94] among the unvaccinated group. Statistically, the difference in prevalence between the vaccinated and unvaccinated participants was not significant [ꭓ^2^ = 0.153, *p* = 0.695]. Similarly, the SARS-CoV-2 Delta variant was identified among 38.89% [95% CI: 20.25–61.64] and 61.11% [95% CI: 38.36–79.75]. Also, the prevalence reported did not differ significantly between the vaccinated and unvaccinated participants [ꭓ^2^ = 1.728, *p* = 0.189] (Fig. 5). However, the prevalence of Omicron related SARS-CoV-2 infection was significantly [ꭓ^2^ = 4.340, *p* = 0.037] higher in unvaccinated participants [72.73%, 95% CI: 42.81–90.08] as compared to vaccinated participants [27.27%, 95% CI: 19.92–57.18] as shown in Fig. 5.

### Association between vaccine type and breakthrough infection

**Fig. 6:**
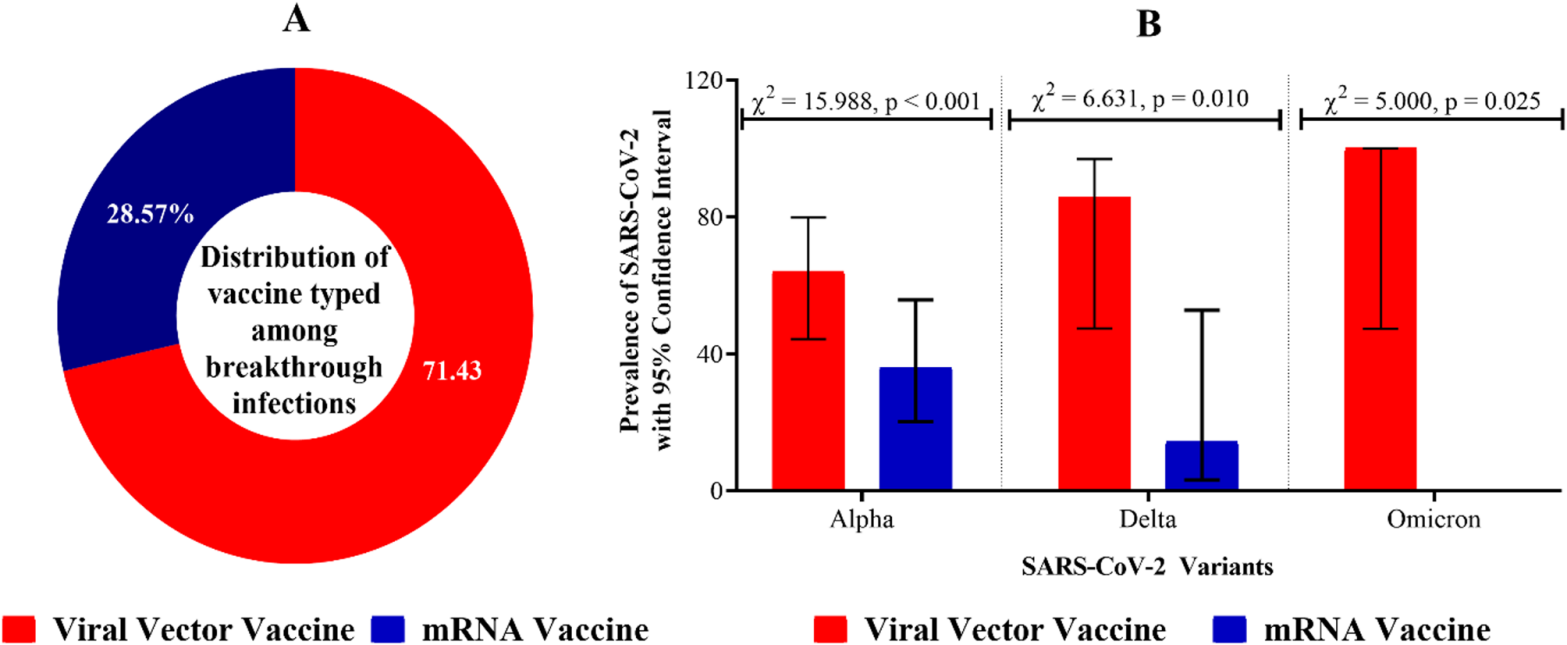
Association of vaccine types with SARS-CoV-2 breakthrough infections. **A** Proportion of COVID-19 vaccine types among individuals with breakthrough infections; **B** Prevalence of SARS-CoV-2 variants (Alpha, Delta, Omicron) among breakthrough infections, stratified by vaccine type. Statistical significance was assessed using the chi-square test (χ2).

Among individuals with SARS-CoV-2 breakthrough infections, 71.43% [95% CI: 54.81–83.65] had received a viral vector vaccine, while 28.57% [95% CI: 16.34–45.19] had received an mRNA vaccine (Fig. 6A). Statistically, the proportion of participants with SARS-CoV-2 breakthrough infection and received vector-based vaccine differed significantly from recipient of mRNA vaccines [χ^2^ = 12.675, *p* < 0.001]. The distribution of SARS-CoV-2 variants among breakthrough infections varied significantly by vaccine type (Fig. 6B). For the Alpha variant, the prevalence among viral vector vaccine recipients [64.00%, 95% CI: 44.33–79.77] was significantly higher compared to mRNA vaccine [36.00%, 95% CI: 20.23–55.67] recipients [χ^2^ = 15.988, *p* < 0.001]. A similar trend was observed for the Delta variant, where viral vector vaccine recipients [85.71%, 95% CI: 47.35–96.81] exhibited a higher prevalence [χ^2^ = 6.631, *p* = 0.010]. Notably, the Omicron variant was detected among only recipients of viral vector vaccine recipients [100.00%, 95% CI: 47.28–100.00] (Fig. 6B).

**Fig. 7:**
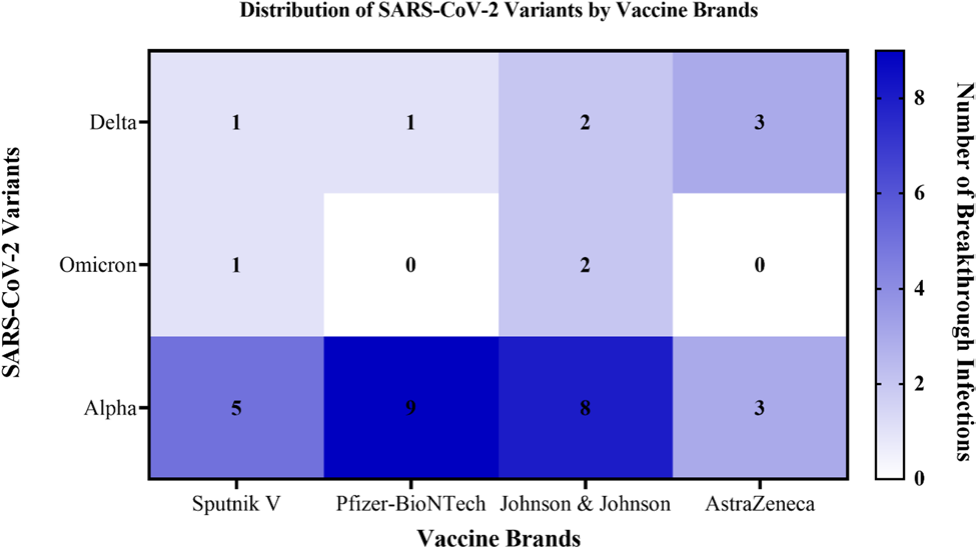
Distribution of SARS-CoV-2 variants by vaccine brands among the study participants

We further examined the distribution of breakthrough infections by vaccine brand and SARS-CoV-2 variants. Figure 7 illustrates the distribution of SARS-CoV-2 variants among participants with breakthrough infections, stratified by vaccine brand. Alpha was the most prevalent variant overall [*n* = 25], with the highest numbers among Pfizer-BioNTech [*n* = 9, 36.00%] and Johnson & Johnson [*n* = 8, 32.00%] recipients. Breakthroughs due to Delta [*n* = 7] occurred across all vaccine types as shown in Fig. 6, while Omicron [*n* = 3] was observed only among Johnson & Johnson recipients [*n* = 2, 66.67%] (Fig. 7). These suggest that breakthrough infections occurred across all vaccine brands, though the low case numbers and uneven distribution preclude definitive comparisons. Detailed description on the distribution of vaccine brand among the participants stratified by age and Ct-value is presented in Table S2.

### Factors associated with breakthrough infection

The study assessed potential predictors of breakthrough SARS-CoV-2 infections using adjusted logistic regression models (Table 5). Demographic characteristics such as sex and age were not significantly associated with breakthrough infections. Specifically, males had lower odds compared to females, but the association was not statistically significant [aOR = 0.706; 95% CI: 0.211–2.361; *p* = 0.572]. Although participants under 20 years had higher crude odds of infection, this was not significant after adjustment [aOR = 0.137; 95% CI: 0.011–1.633; *p* = 0.116]. Symptomatic presentation overall did not predict breakthrough infection [aOR = 0.757; 95% CI: 0.108–3.311; *p* = 0.779] (Table 5). However, specific symptoms were significantly associated. Diarrhoea was associated with markedly increased odds of breakthrough infection [aOR = 9.668; 95% CI: 2.192–15.759; *p* = 0.022]. Sore throat [aOR = 8.991; 95% CI: 3.129–10.981; *p* = 0.038] and joint pain [aOR = 10.156; 95% CI: 1.051–12.372; *p* = 0.039] were also significantly associated (Table 5). Cycle threshold (Ct) values were significantly associated with breakthrough SARS-CoV-2 infections. Compared to individuals with Ct values < 25, participants with Ct values between 25 and 30 had increased odds of breakthrough infection [aOR = 11.327; 95% CI: 2.716–15.772; *p* = 0.012] (Table 5). Similarly, Ct values > 30 were also associated with higher odds of breakthrough infection [aOR = 4.010; 95% CI: 1.906–26.760; *p* = 0.047]. Regarding variant type, infection with the Omicron variant was significantly associated with breakthrough infection compared to the Alpha variant [aOR = 4.383; 95% CI: 1.213–12.585; *p* = 0.034]. No significant association was observed for the Delta variant [aOR = 0.667; 95% CI: 0.149–2.997; *p* = 0.598] (Table 5).

**Table 5:** Logistic regression analysis of factors associated with breakthrough infections within the Accra metropolis

Variables	Crude			Adjusted		
	OR	95% CI	*p*–value	OR	95% CI	*p*–value
Gender						
Male	1.416	0.424–4.736	0.572	0.706	0.211–2.361	0.572
Female (ref)	*1*	*1*		*1*	–	–
Age (years)						
< 20	5.478	1.731–10.029	0.098	0.137	0.011–1.633	0.116
21–40	1.190	0.312–4.536	0.799	0.750	0.104–2.432	0.776
41–60	2.945	0.455–4.276	0.257	0.381	0.157–2.559	0.321
> 60 (ref)	*1*	–	–	*1*	–	–
Clinical Presentation						
Symptomatic	1.322	0.188–3.276	0.677	0.757	0.108–3.311	0.779
Asymptomatic (ref)	*1*	–	–	*1*	–	–
Symptoms Presented						
Chills: Yes	1.268	0.510–3.156	0.607	3.316	1.233–10.428	**0.046**
Chills: No (ref)	*1*	–	–	*1*	–	–
Cough: Yes	1.660	0.946–2.913	0.062	0.316	0.038–2.603	0.284
Cough: No (ref)	*1*	–	–	*1*	–	–
Diarrhoea: Yes	2.727	0.304–8.270	0.004	9.668	2.192–15.759	**0.022**
Diarrhoea: No (ref)	*1*	–	–	*1*	–	–
Fever > 38℃: Yes	4.565	1.576–6.209	0.106	0.664	0.041–4.674	0.664
Fever > 38℃: No (ref)	*1*	–	–	*1*	–	–
Sore Throat: Yes	1.099	0.531–2.275	**0.046**	8.991	3.129–10.981	**0.038**
Sore Throat: No (ref)	*1*	–	–	*1*	–	–
Headache: Yes	1.902	0.822–4.400	0.116	10.156	1.051–12.372	**0.039**
Headache: No (ref)	*1*	–	–	*1*	–	–
Joint Pain: Yes	1.324	0.068–5.640	0.844	0.872	0.403–4.915	0.931
Joint Pain: No (ref)	*1*	–	–	*1*	–	–
Muscle Pain: Yes	0.984	0.235–3.881	0.984	0.097	0.040–2.627	0.166
Muscle Pain: No (ref)	*1*	–	–	*1*	–	–
Runny Nose: Yes	0.249	0.051–1.106	0.072	0.372	0.004–2.759	0.673
Runny Nose: No (ref)	*1*	–	–	*1*	–	–
Body Weakness: Yes	1.333	0.200–8.819	0.779	3.316	1.233–10.428	**0.046**
Body Weakness: No (ref)	*1*	–	–	*1*	–	–
Severity of Infection						
Ct–value: 25–30	2.430	0.792–7.377	**0.045**	11.327	2.716–15.772	**0.012**
Ct–value: >30	1.789	0.709–4.290	0.236	4.01	1.906–26.760	**0.047**
Ct–value: < 25	*1*	–	–	*1*	–	–
SARS-CoV-2 Variants						
Delta	0.796	0.297–2.184	0.675	0.667	0.149–2.997	0.598
Omicron	0.445	0.121–1.691	0.251	4.383	1.213–12.585	**0.034**
Alpha	*1*	–	–	*1*	–	–

p-values < 0.05 are deemed statistically significant

*1 *Reference category (ref), *OR* Odds ratio, *Ct-value* Cycle threshold value

## Discussion

This study investigated the prevalence, clinical characteristics, viral load profiles, and variant distribution of SARS-CoV-2 breakthrough infections in the Accra Metropolis, comparing vaccinated and unvaccinated individuals. The analysis incorporated cycle threshold (Ct) values as indicators of viral burden, assessed symptom patterns, and examined the distribution of variants among infection cases. The findings support the protective effect of vaccination against severe disease [22] and suggest differences in viral replication and symptom presentation based on vaccination status.

SARS-CoV-2 infection was confirmed in individuals who had received at least one dose of a COVID-19 vaccine (Table 2). Although this proportion reflects the occurrence of breakthrough infections, it does not necessarily imply vaccine failure. Breakthrough cases have been widely reported globally, particularly with the emergence of variants that exhibit partial immune escape [23]. The rate observed in this study aligns with reports from both resource-rich and resource-limited settings, where breakthrough infections have been documented in the context of widespread vaccine rollout and ongoing community transmission [24, 25].

Importantly, symptomatic cases were significantly prevalent among unvaccinated individuals [67.44%] (Table 2), supporting the role of vaccination in reducing clinical severity. These findings are consistent with published evidence indicating that vaccination, even when it does not prevent infection, contributes to milder disease outcomes, with lower rates of hospitalisation and mortality [22, 26]. The higher rate of asymptomatic or mildly symptomatic presentation in the vaccinated group is likely a consequence of vaccine-induced immune priming that moderates the host response to infection [27].

Age-stratified analysis indicated that younger participants and middle-aged (41–60 years) individuals were more frequently unvaccinated (Table 2). Several factors may account for this, including delayed vaccine access during the initial phases of the vaccination campaign, lower perceived personal risk, and varying levels of vaccine hesitancy [28, 29]. Although no significant difference in vaccination status was found among older adults (>60 years), interpretation is limited by the relatively small sample size within this subgroup. Nonetheless, the general pattern may reflect broader demographic trends in vaccine uptake.

Viral load, as estimated by Ct values, differed significantly between the groups (Fig. 2A). Vaccinated individuals were less likely to have Ct values below 25, which are typically associated with higher viral replication and potentially greater infectiousness. Specifically, the frequency of low Ct values was significantly reduced among vaccinated individuals (Fig. 2A), consistent with other reports showing that vaccination reduces the magnitude of viral replication during acute infection [30, 31]. This observation aligns with the immunological principle that vaccine-primed responses, although not always sterilising, can limit the extent and duration of viral replication [32, 33].

Further analysis revealed that median Ct values for the N and E gene targets were significantly higher in the vaccinated group, suggesting lower overall viral burden (Fig. 2B). Although the RdRP gene also showed a similar trend, the difference did not reach statistical significance. The findings support the view that, in addition to mitigating disease severity, vaccination may reduce transmissibility by limiting the amount of virus shed [34]. Notably, Ct values for RdRP and E genes showed a negative correlation with time since vaccination, implying waning immunity. This observation strengthens the rationale for booster doses, particularly in high-risk populations, to maintain adequate immune protection over time [35, 36].

More so, a clear inverse relationship was observed between the time since the last vaccine dose and viral load across all three gene targets (Fig. 3). Individuals who tested positive at longer intervals post-vaccination tended to have higher estimated viral loads, as shown by strong negative correlations, particularly for the RdRP and E genes (Fig. 3). These results are consistent with existing data suggesting a gradual reduction in vaccine-induced immune protection over time [37, 38]. Such findings support the biological plausibility that as immune responses wane, the ability to limit viral replication diminishes. In countries where booster coverage remains limited, including many parts of sub-Saharan Africa, this pattern reinforces the importance of maintaining adequate immune protection through the administration of booster doses of the vaccine, especially for individuals at increased risk of severe disease.

We considered the possibility that the inclusion of asymptomatic cases may have influenced the observed association between vaccination status and viral load, as asymptomatic individuals could be detected at later stages of infection when viral loads are lower. Our stratified analysis (Fig. 4) demonstrated that symptomatic participants were significantly presented with Ct values < 25, indicative of higher viral loads, whereas asymptomatic participants were more frequently represented in higher Ct categories. This distribution aligns with the biological expectation that symptomatic presentation is often associated with active viral replication [39, 40]. Importantly, when analyses were stratified by symptom status, the relationship between vaccination status and Ct values remained consistent (Fig. 3), indicating that symptom status did not act as a major confounder. Nevertheless, given that Ct values were measured at a single diagnostic time point, some residual confounding due to variability in the timing of detection cannot be entirely excluded as also emphasized by other studies [41].

Beyond reinforcing the need for boosters, the observed inverse correlation between time since vaccination and viral load offers a practical, real-time proxy for assessing waning vaccine effectiveness in clinical or surveillance settings. In LMICs with limited access to serological or immunogenicity monitoring, such Ct-derived viral load trends could serve as operational markers for scheduling or prioritizing booster campaigns. This approach is particularly useful for identifying risk windows, for instance after 60 days post-vaccination, where higher viral loads may re-emerge, potentially signalling reduced protection. Integrating this parameter into national vaccine policy planning may support more efficient resource allocation and targeted protection strategies.

The pattern of symptom also varied by vaccination status. While the general symptomatic status did not significantly predict breakthrough infections (Table 5), certain symptoms were more common in vaccinated individuals who experienced breakthrough infections. Diarrhoea, sore throat, and joint pain were more common in this group, while unvaccinated individuals more often presented with headache and typical respiratory symptoms such as cough, and nasal congestion (Table 2). This pattern suggests that vaccination may influence the clinical manifestation of SARS-CoV-2 infection. Emerging evidence indicates that immune priming through vaccination may alter host response dynamics, potentially leading to a shift in symptomatology towards non-respiratory or systemic features [42, 43]. Previous studies have similarly observed atypical symptom profiles in vaccinated individuals, including a higher prevalence of gastrointestinal or musculoskeletal complaints [44, 45]. The increased reporting of symptoms such as diarrhoea and joint pain in vaccinated individuals may reflect a genuine shift in clinical phenotype due to changes in immune activation pathways or viral tissue tropism. Alternatively, reporting bias cannot be excluded, as vaccinated individuals may be more likely to report milder or non-specific symptoms during diagnostic encounters. Further investigation in larger cohorts and across diverse vaccine platforms is warranted to clarify whether these trends are consistent and biologically driven.

Three major SARS-CoV-2 variants, the Alpha, Delta, and Omicron, were detected during the study period [12]. While Alpha and Delta were distributed relatively evenly between vaccinated and unvaccinated groups, Omicron infections were more frequently detected in unvaccinated individuals (Fig. 5). As at 2022, when the COVID-19 pandemic was ending, Omicron was the most dominant COVID-19 variant [46]. Thus, it is not surprising that omicron was frequently reported among the unvaccinated cohort. This pattern contrasts with global observations in which Omicron has been associated with a substantial proportion of breakthrough infections due to its extensive spike protein mutations that confer partial immune escape [25, 47]. The observed discrepancy may reflect differences in variant circulation timelines, vaccination coverage, or population-level exposure patterns during the sampling period [48]. While this study was conducted in a single urban centre, the epidemiological patterns observed such as increased breakthrough infections due to Omicron and waning vaccine-induced protection are consistent with broader trends reported in other LMICs [49, 50]. However, study in the Maryland, USA, reported that a significant proportion of breakthrough infections were associated with Delta [51]. Given the widespread use of similar vaccines such as Oxford-AstraZeneca and delays in booster implementation across many African and Southeast Asian countries, these findings likely reflect a wider regional experience. More so, multivariable logistic regression analysis indicated that infection with the Omicron variant was significantly associated with breakthrough status (Table 5), suggesting that, although the absolute number of Omicron cases was higher in the unvaccinated group, a substantial proportion of breakthrough infections were still attributable to Omicron. This supports existing laboratory and clinical studies showing that Omicron and its sub-lineages can partially evade vaccine-induced immunity, particularly in individuals who have not yet received booster doses [43, 48].

Further analysis revealed that the type of vaccine received was significantly associated with breakthrough infections (Fig. 6A). Individuals who had received viral vector vaccines accounted for a larger proportion of breakthrough cases compared to those vaccinated with mRNA vaccines. Specifically, 71.43% of the breakthrough infections occurred in recipients of viral vector vaccines, with only 28.57% occurring among those who had received mRNA vaccines (Fig. 6A). The difference was statistically significant, suggesting a variation in protection against infection between the two vaccine platforms during the study period and consistent with report from Nepal [25].

Notably, variant-specific distributions among breakthrough cases also differed by vaccine platform (Fig. 6B). Alpha, Delta, and Omicron variants were all significantly more prevalent among viral vector vaccine recipients. In particular, Omicron infections were observed exclusively in individuals who had received viral vector vaccines. These findings are consistent with published reports showing that viral vector vaccines, while effective at preventing severe disease, may produce lower levels of neutralising antibodies compared to mRNA vaccines [52, 53]. This difference in immune response may be more apparent in the presence of variants with multiple mutations, such as Omicron, which are known to reduce the effectiveness of vaccine-induced immunity over time.

Although the Alpha variant was the most frequently detected across all vaccines, its distribution appeared higher among Pfizer-BioNTech and Johnson & Johnson recipients (Fig. 7). Delta variant infections were relatively fewer but occurred mainly in AstraZeneca and Johnson & Johnson recipients, while Omicron infections were observed in only three cases across Sputnik V and Johnson & Johnson (Fig. 7). These findings are consistent with other studies that reported breakthrough infections across multiple vaccine types without clear evidence of variant-specific clustering by brand [24, 54, 55]. More so, the data suggest that while breakthrough infections occurred across all vaccine platforms, there was no strong evidence to implicate any single vaccine brand as disproportionately vulnerable to a particular variant in this study population. This further reinforces the notion that breakthrough infections are less a reflection of vaccine brand and more a function of the interplay between waning immunity, the characteristics of circulating variants, and the timing of exposure.

It is worth noting that viral vector vaccines including Oxford-AstraZeneca and Johnson & Johnson, formed the basis of the national COVID-19 vaccination programme in Ghana. mRNA vaccines were available only in limited quantities, primarily through donations [56]. This context likely influenced the pattern of breakthrough infections observed in the study. While direct comparisons of vaccine effectiveness were not the primary aim of this analysis, the findings point to the importance of considering vaccine platform when interpreting infection trends, especially where multiple vaccine types have been used. The pattern observed may reflect differences in the immune response generated by these vaccines, as well as the interval since the last dose. These results support ongoing discussions around booster vaccination, especially the use of mRNA vaccines as additional doses in populations initially immunised with viral vector vaccines. Monitoring infection trends in relation to vaccine type remains important, particularly as SARS-CoV-2 variants continue to circulate and evolve [57, 58].

Looking forward, novel vaccine platforms are being developed to address the challenges of immune escape and waning protection observed with current vaccines. Xie et al. [59] described new approaches such as multivalent and pan-sarbecovirus vaccines, which aim to provide broader and more durable immunity across variants. While these next-generation vaccines are not yet widely available in LMICs, they hold promise for mitigating breakthrough infections and could play an important role in strengthening long-term pandemic preparedness in regions like sub-Saharan Africa.

Cycle threshold (Ct) values emerged as strong predictors of breakthrough infection (Table 5). Individuals with Ct values above 25 were significantly more likely to have been vaccinated. Since higher Ct values generally correspond to lower viral loads, these results reinforce the interpretation that vaccination reduces viral replication even in the context of breakthrough infection [22]. This has important implications for transmission dynamics, as lower viral loads are associated with reduced transmissibility [30, 60]. Moreover, these findings validate the use of Ct values as a useful, albeit indirect, marker for assessing disease severity and transmission potential in epidemiological surveillance.

The use of Ct values as a proxy for viral burden is especially useful in settings where quantitative viral load testing or genomic sequencing is not routinely available. This study introduces a scalable, semi-quantitative RT-PCR–based framework for variant surveillance and viral load estimation that can be integrated into routine diagnostics in LMICs lacking genomic capacity. Many LMICs rely on qualitative RT-PCR assays without the capacity for routine sequencing, making Ct-based epidemiological inference a practical alternative. Our approach and findings may therefore inform surveillance strategies in other under-resourced settings where diagnostic capacity is limited.

An important aspect that warrants consideration is the long-term effect of SARS-CoV-2 infection on immune function. Recent studies have shown that individuals recovering from acute infection may experience sustained immune dysregulation, characterised by persistent inflammatory activity, impaired T-cell responses, and alterations in antibody production [61, 62]. These changes could influence susceptibility to reinfection and may also compromise the durability of vaccine-induced protection. Although the present study focused on acute infection and viral load dynamics, the evidence of ongoing immune alterations after COVID-19 demonstrates the need to integrate both short- and long-term immune consequences into vaccination strategies and booster policies. This is particularly relevant for resource-limited settings such as Ghana, where maintaining both immediate and long-term protection against SARS-CoV-2 remains a public health priority.

This study provides evidence that COVID-19 vaccination reduces both disease severity and viral load among infected individuals in an urban Ghanaian setting. It also points to distinct symptomatic patterns and variant distributions associated with vaccination status. The findings emphasize the relevance of vaccination, including booster campaigns, in mitigating the public health burden of SARS-CoV-2, especially in settings with ongoing community transmission and variant evolution.

### Limitations of the study

This study has some limitations that warrant consideration. Although the total sample size [*n* = 81] was adequate for the primary analyses, including logistic regression, some subgroup comparisons, particularly symptom-specific and age-stratified analyses were based on small sample sizes. As such, findings from these subgroups should be interpreted with caution due to the potential for reduced statistical power.

Cycle threshold (Ct) values, while informative as a proxy for viral load, are subject to variability across RT-PCR platforms and protocols. To minimise this, all testing in our study was performed using a single assay system with internal synthetic RNA controls and run-to-run consistency maintained. Nonetheless, minor fluctuations inherent to PCR-based detection cannot be entirely excluded.

Variant identification was conducted using mutation-specific RT-PCR assays, which are a practical and validated method for epidemiological surveillance. However, they do not provide the full resolution of whole genome sequencing, and limits the possibility of the identification of the sub lineage of the variants identified.

Additionally, while key confounding variables such as age and vaccination status were considered in multivariable models, other factors such as prior SARS-CoV-2 exposure, comorbidities, or time from symptom onset to sample collection were not available and may have influenced Ct values or clinical presentation.

Finally, the retrospective design based on data from a cross-sectional design limits causal inference. The findings reflect associations observed at a specific time point and may not capture the evolving dynamics of host immunity, viral variants, or vaccine effectiveness over time. Despite these limitations, the study provides valuable real-world data from a sub-Saharan African setting and contributes to the understanding of SARS-CoV-2 breakthrough infections in underrepresented populations.

## Conclusion

This study found that individuals with SARS-CoV-2 breakthrough infections had significantly higher cycle threshold (Ct) values than those who were unvaccinated, indicating lower viral loads among the vaccinated. The Omicron variant was more frequently associated with breakthrough infections, consistent with its capacity for partial immune evasion. These results support the need for high vaccine uptake and timely administration of booster doses. This will reduce viral replication and transmission The findings, thus reaffirm the importance of Ct values as a practical measure of viral burden, particularly in settings where more advanced quantification methods are not routinely available. Thus, sustained genomic and Ct-based monitoring remains essential to tracking variant circulation and guides adaptive public health interventions, not only in Ghana but also in other LMICs facing similar diagnostic and immunisation constraints.

## Supplementary Information

Supplementary Material 1

Supplementary Material 2

Supplementary Material 3

## Data Availability

All relevant data underlying the findings of this study are contained within the manuscript and its Supporting Information files. A fully de-identified dataset has been deposited in Zenodo and is accessible at https:/doi.org/10.5281/zenodo.15576531. The dataset includes the following variables: participant age, sex, COVID-19 vaccination status, vaccine type, days since last vaccine dose, presence of symptoms (binary), individual symptom profiles, RT-PCR cycle threshold (Ct) values and estimated viral load for the N, RdRP, and E genes, and SARS-CoV-2 variant type (Alpha, Delta, or Omicron). A detailed codebook explaining all variables, categorical definitions, and coding schemes is provided in Table S3.

## References

[1] CDC. COVID-19 vaccine breakthrough infections reported to CDC — United States, January 1–April 30, 2021. MMWR Morb Mortal Wkly Rep. 2021;70:792–3. 10.15585/mmwr.mm7021e3. 34043615 10.15585/mmwr.mm7021e3 PMC8158893

[2] Feikin DR, Higdon MM, Abu-Raddad LJ, Andrews N, Araos R, Goldberg Y, et al. Duration of effectiveness of vaccines against SARS-CoV-2 infection and COVID-19 disease: results of a systematic review and meta-regression. Lancet. 2022;399:924–44. 10.1016/S0140-6736(22)00152-0. 35202601 10.1016/S0140-6736(22)00152-0 PMC8863502

[3] de Souza AS, de Freitas Amorim VM, Guardia GDA, dos Santos FF, Ulrich H, Galante PAF, et al. Severe acute respiratory syndrome coronavirus 2 variants of concern: a perspective for emerging more transmissible and vaccine-resistant strains. Viruses. 2022;14:827. 10.3390/v14040827. 35458557 10.3390/v14040827 PMC9029021

[4] Haque A, Pant AB. Mitigating covid-19 in the face of emerging virus variants, breakthrough infections and vaccine hesitancy. J Autoimmun. 2022;127:102792. 10.1016/j.jaut.2021.102792. 34995958 10.1016/j.jaut.2021.102792 PMC8719928

[5] McCormick KD, Jacobs JL, Mellors JW. The emerging plasticity of SARS-CoV-2. Science. 2021;371:1306–8. 10.1126/science.abg4493. 33766871 10.1126/science.abg4493

[6] Abdulrahman A, Mallah SI, Alawadhi A, Perna S, Janahi EM, AlQahtani MM. Association between RT-PCR Ct values and COVID-19 new daily cases: a multicenter cross-sectional study. Infez Med. 2021;29:416–26. 10.53854/liim-2903-13. 35146347 10.53854/liim-2903-13 PMC8805503

[7] Bouzid D, Vila J, Hansen G, Manissero D, Pareja J, Rao SN et al. Systematic review on the association between respiratory virus real-time PCR cycle threshold values and clinical presentation or outcomes. J Antimicrob Chemother. 2021;76 Supplement_3:iii33–49. 10.1093/jac/dkab246. 10.1093/jac/dkab246 PMC8460103 34555159

[8] Acharya CB, Schrom J, Mitchell AM, Coil DA, Marquez C, Rojas S, et al. Viral load among vaccinated and unvaccinated, asymptomatic and symptomatic persons infected with the SARS-CoV-2 delta variant. Open Forum Infect Dis. 2022;9:ofac135. 10.1093/ofid/ofac135. 35479304 10.1093/ofid/ofac135 PMC8992250

[9] Andeweg SP, van de Kassteele J, Wang X, van Maarseveen N, Vlaemynck B, Bos S, et al. Estimating the effect of COVID-19 vaccination and prior infection on cycle threshold values as a proxy of SARS-CoV-2 viral load. Int J Infect Dis. 2025;153:107362. 10.1016/j.ijid.2024.107362. 39662741 10.1016/j.ijid.2024.107362

[10] Sala E, Shah IS, Manissero D, Juanola-Falgarona M, Quirke A-M, Rao SN. Systematic review on the correlation between SARS-CoV-2 real-time PCR cycle threshold values and epidemiological trends. Infect Dis Ther. 2023;12:749–75. 10.1007/s40121-023-00772-7. 36811776 10.1007/s40121-023-00772-7 PMC9945817

[11] Hyug Choi J, Sook Jun M, Yong Jeon J, Kim H-S, Kyung Kim Y, Ho Jeon C, et al. Global lineage evolution pattern of SARS-CoV-2 in Africa, America, Europe, and Asia: a comparative analysis of variant clusters and their relevance across continents. J Transl Intern Med. 2023;11:410–22. 10.2478/jtim-2023-0118. 10.2478/jtim-2023-0118 PMC10732492 38130632

[12] Aboagye FT, Annison L, Hackman HK, Acquah ME, Ashong Y, Owusu-Frimpong I, et al. Molecular epidemiology of SARS-CoV-2 within Accra metropolis postlockdown. Adv Virol. 2024;2024:e2993144. 10.1155/2024/2993144. 10.1155/2024/2993144 PMC10997420 38584794

[13] George A, Murugan T, Sampath S, N SM. Epidemiology of COVID-19 and the utility of cycle threshold (Ct) values in predicting the severity of disease. Cureus. 2023;15:e43679. 10.7759/cureus.43679. 37724229 10.7759/cureus.43679 PMC10505265

[14] Natarajan S, Ranganathan M, Natarajan PL, Nesakumar M, Anbalagan S, Lucia Precilla K, et al. Comparison of real-time RT-PCR cycle threshold (Ct) values with clinical features and severity of COVID-19 disease among hospitalized patients in the first and second waves of COVID-19 pandemic in Chennai, India. Journal of Clinical Virology Plus. 2023;3:100146. 10.1016/j.jcvp.2023.100146. 37016620 10.1016/j.jcvp.2023.100146 PMC10043973

[15] CDC. Interim Guidelines for Collecting and Handling of Clinical Specimens for COVID-19 Testing. Centers for Disease Control and Prevention. 2020. https://www.cdc.gov/coronavirus/2019-ncov/lab/guidelines-clinical-specimens.html. Accessed 25 Jan 2024.

[16] Shrestha LB, Pokharel K. Standard operating procedure for specimen collection, packaging and transport for diagnosis of SARS-COV-2. JNMA J Nepal Med Assoc. 2020;58:627. 32968304 10.31729/jnma.5260 PMC7580376

[17] Aboagye FT, Acquah ME. Isolation and amplification of SARS-CoV-2 RNA from nasopharyngeal specimen. Protoc Io. 2023;1–7. 10.17504/protocols.io.q26g7y32kgwz/v1.

[18] Liotti FM, Menchinelli G, Marchetti S, Morandotti GA, Sanguinetti M, Posteraro B, et al. Evaluation of three commercial assays for SARS-CoV-2 molecular detection in upper respiratory tract samples. Eur J Clin Microbiol Infect Dis. 2021;40:269–77. 10.1007/s10096-020-04025-0. 32885293 10.1007/s10096-020-04025-0 PMC7471581

[19] Umunnakwe CN, Makatini ZN, Maphanga M, Mdunyelwa A, Mlambo KM, Manyaka P, et al. Evaluation of a commercial SARS-CoV-2 multiplex PCR genotyping assay for variant identification in resource-scarce settings. PLoS ONE. 2022;17:e0269071. 35749403 10.1371/journal.pone.0269071 PMC9231807

[20] Liotti FM, De Maio F, Ippoliti C, Santarelli G, Monzo FR, Sali M, et al. Two-period study results from a large Italian hospital laboratory attesting SARS-CoV-2 variant PCR assay evolution. Microbiol Spectr. 2022;10:e02922-22. 10.1128/spectrum.02922-22. 36409091 10.1128/spectrum.02922-22 PMC9769628

[21] Nielsen MC, Machado RRG, Mitchell BM, McConnell AJ, Saada NI, Weaver SC, et al. A comparison of seegene technologies Novaplex SARS-CoV-2 variants I, II, and IV assays with Spike gene sequencing for detection of known severe acute respiratory syndrome coronavirus 2 variants. J Mol Diagn. 2022;24(5):455–61. 10.1016/j.jmoldx.2022.02.001. 35218945 10.1016/j.jmoldx.2022.02.001 PMC8865930

[22] Chan L, Pinedo K, Stabile MA, Hamlin RE, Pienkos SM, Ratnasiri K, et al. Prior vaccination prevents overactivation of innate immune responses during COVID-19 breakthrough infection. Sci Transl Med. 2025;17(783):eadq1086. 10.1126/scitranslmed.adq1086. 39879318 10.1126/scitranslmed.adq1086 PMC12142493

[23] Yu X, Juraszek J, Rutten L, Bakkers MJG, Blokland S, Melchers JM, et al. Convergence of immune escape strategies highlights plasticity of SARS-CoV-2 Spike. PLoS Pathog. 2023;19:e1011308. 10.1371/journal.ppat.1011308. 37126534 10.1371/journal.ppat.1011308 PMC10174534

[24] Aga AM, Mulugeta D, Gebreegziabxier A, Zeleke GT, Girmay AM, Tura GB, et al. Genome diversity of SARS-CoV-2 lineages associated with vaccination breakthrough infections in Addis Ababa, Ethiopia. BMC Infect Dis. 2025;25:738. 10.1186/s12879-025-11107-x. 40410660 10.1186/s12879-025-11107-x PMC12102866

[25] Karmacharya A, Rai K, Siwakoti S, Khanal B, Bhattarai NR. COVID-19 breakthrough infections in vaccinated individuals at BPKIHS, Nepal. BMC Infect Dis. 2024;24:1003. 10.1186/s12879-024-09902-z. 39300352 10.1186/s12879-024-09902-z PMC11411789

[26] Maleki B, Sadeghian AM, Ranjbar M. Impact of vaccination against SARS-CoV-2 on mortality risk, ICU admission rate, and hospitalization length in hospitalized COVID-19 patients: a cross-sectional study. BMC Infect Dis. 2025;25:144. 10.1186/s12879-025-10530-4. 39885405 10.1186/s12879-025-10530-4 PMC11783754

[27] Duan R, Mao Q, Ding X, Qiu Q, Wang P. Immunologic features of asymptomatic postvaccination infections with the delta variant of SARS-CoV‐2 in adults. Immun Inflamm Dis. 2022;10:e670. 10.1002/iid3.670. 35759224 10.1002/iid3.670 PMC9210569

[28] Frempong CS, Tarkang EE, Amu H, Gborglah M, Salu S, Otoo DM. Prevalence and factors associated with COVID-19 vaccine hesitancy among adults in Ghana: a population-based cross-sectional study in the Volta region. Discov Public Health. 2024;21:126. 10.1186/s12982-024-00236-4.

[29] McCarthy RNE, Donkoh ET, Arthur DD, Dassah ET, Boadu KO, Otoo JE, et al. Public relations strategies employed by the Ghana health service to address COVID-19 vaccine hesitancy: a qualitative inquiry. Trop Med Health. 2023;51:26. 10.1186/s41182-023-00519-7. 37170342 10.1186/s41182-023-00519-7 PMC10175053

[30] Fryer HR, Golubchik T, Hall M, Fraser C, Hinch R, Ferretti L, et al. Viral burden is associated with age, vaccination, and viral variant in a population-representative study of SARS-CoV-2 that accounts for time-since-infection-related sampling bias. PLoS Pathog. 2023;19:e1011461. 10.1371/journal.ppat.1011461. 37578971 10.1371/journal.ppat.1011461 PMC10449197

[31] Levine-Tiefenbrun M, Yelin I, Katz R, Herzel E, Golan Z, Schreiber L, et al. Initial report of decreased SARS-CoV-2 viral load after inoculation with the BNT162b2 vaccine. Nat Med. 2021;27:790–2. 33782619 10.1038/s41591-021-01316-7

[32] Jangra S, De Vrieze J, Choi A, Rathnasinghe R, Laghlali G, Uvyn A, et al. Sterilizing immunity against SARS-CoV‐2 infection in mice by a single‐shot and lipid amphiphile imidazoquinoline TLR7/8 agonist‐adjuvanted recombinant spike protein vaccine. Angew Chem Int Ed. 2021;60:9467–73. 10.1002/anie.202015362. 10.1002/anie.202015362 PMC8014308 33464672

[33] Rutigliano JA, Morris MY, Yue W, Keating R, Webby RJ, Thomas PG, et al. Protective memory responses are modulated by priming events prior to challenge. J Virol. 2010;84:1047–56. 10.1128/JVI.01535-09. 19889782 10.1128/JVI.01535-09 PMC2798378

[34] Garcia-Knight M, Anglin K, Tassetto M, Lu S, Zhang A, Goldberg SA, et al. Infectious viral shedding of SARS-CoV-2 delta following vaccination: a longitudinal cohort study. PLoS Pathog. 2022;18:e1010802. 10.1371/journal.ppat.1010802. 36095030 10.1371/journal.ppat.1010802 PMC9499220

[35] Burckhardt RM, Dennehy JJ, Poon LLM, Saif LJ, Enquist LW. Are COVID-19 vaccine boosters needed? The science behind boosters. J Virol. 2022;96:e01973–21. 10.1128/jvi.01973-21. 34817198 10.1128/jvi.01973-21 PMC8827020

[36] Meng H, Mao J, Ye Q. Booster vaccination strategy: necessity, immunization objectives, immunization strategy, and safety. J Med Virol. 2022;94:2369–75. 10.1002/jmv.27590. 35028946 10.1002/jmv.27590

[37] Goel RR, Painter MM, Apostolidis SA, Mathew D, Meng W, Rosenfeld AM, et al. mRNA vaccines induce durable immune memory to SARS-CoV-2 and variants of concern. Science. 2021;374:abm0829. 10.1126/science.abm0829. 34648302 10.1126/science.abm0829 PMC9284784

[38] Pooley N, Abdool Karim SS, Combadière B, Ooi EE, Harris RC, El Guerche Seblain C, et al. Durability of vaccine-induced and natural immunity against COVID-19: a narrative review. Infect Dis Ther. 2023;12:367–87. 10.1007/s40121-022-00753-2. 36622633 10.1007/s40121-022-00753-2 PMC9828372

[39] Zou L, Ruan F, Huang M, Liang L, Huang H, Hong Z, et al. SARS-CoV-2 viral load in upper respiratory specimens of infected patients. N Engl J Med. 2020;382:1177–9. 10.1056/NEJMc2001737. 32074444 10.1056/NEJMc2001737 PMC7121626

[40] Marks M, Millat-Martinez P, Ouchi D, Roberts CH, Alemany A, Corbacho-Monné M, et al. Transmission of COVID-19 in 282 clusters in Catalonia, Spain: a cohort study. Lancet Infect Dis. 2021;21:629–36. 10.1016/S1473-3099(20)30985-3. 33545090 10.1016/S1473-3099(20)30985-3 PMC7906723

[41] Hay JA, Kennedy-Shaffer L, Kanjilal S, Lennon NJ, Gabriel SB, Lipsitch M, et al. Estimating epidemiologic dynamics from cross-sectional viral load distributions. Science. 2021;373:eabh0635. 10.1126/science.abh0635. 34083451 10.1126/science.abh0635 PMC8527857

[42] Almalki OS, Santali EY, Alhothali AA, Ewis AA, Shady A, Fathelrahman AI, et al. The role of blood groups, vaccine type and gender in predicting the severity of side effects among university students receiving COVID-19 vaccines. BMC Infect Dis. 2023;23:378. 10.1186/s12879-023-08363-0. 37280542 10.1186/s12879-023-08363-0 PMC10242595

[43] Shahbaz S, Bozorgmehr N, Lu J, Osman M, Sligl W, Tyrrell DL, et al. Analysis of SARS-CoV-2 isolates, namely the Wuhan strain, delta variant, and Omicron variant, identifies differential immune profiles. Microbiol Spectr. 2023;11:e01256–23. 10.1128/spectrum.01256-23. 37676005 10.1128/spectrum.01256-23 PMC10581158

[44] Adly HM, Saleh SAK, Garout MA, Abdulkhaliq AA, Khafagy AA, Saati AA, et al. Post COVID-19 symptoms among infected vaccinated individuals: a cross-sectional study in Saudi Arabia. J Epidemiol Glob Health. 2023;13:740–50. 10.1007/s44197-023-00146-9. 37665505 10.1007/s44197-023-00146-9 PMC10686930

[45] Canas LS, Österdahl MF, Deng J, Hu C, Selvachandran S, Polidori L, et al. Disentangling post-vaccination symptoms from early COVID-19. eClinicalMedicine. 2021. 10.1016/j.eclinm.2021.101212. 34873584 10.1016/j.eclinm.2021.101212 PMC8635464

[46] Daria S, Islam MR. The SARS-CoV‐2 omicron wave is indicating the end of the pandemic phase but the COVID‐19 will continue. J Med Virol. 2022;94:2343–5. 10.1002/jmv.27635. 35098543 10.1002/jmv.27635 PMC9015536

[47] Kandeel A, Moatasim Y, Fahim M, Bahaaeldin H, El-Shesheny R, Roshdy WH, et al. Comparison of SARS-Cov-2 omicron variant with the previously identified SARS-Cov-2 variants in Egypt, 2020–2022: insight into SARS-Cov-2 genome evolution and its impact on epidemiology, clinical picture, disease severity, and mortality. BMC Infect Dis. 2023;23:542. 10.1186/s12879-023-08527-y. 37596534 10.1186/s12879-023-08527-y PMC10439637

[48] Dadonaite B, Burrell AR, Logue J, Chu HY, Payne DC, Haslam DB, et al. SARS-CoV-2 neutralizing antibody specificities differ dramatically between recently infected infants and immune-imprinted individuals. J Virol. 2025;99:e00109–25. 10.1128/jvi.00109-25. 40130874 10.1128/jvi.00109-25 PMC11998527

[49] Pandit P, Bhatt P, Sahay RR, Joshi Y, Patil DY, Yadav PD. A case of breakthrough infection with SARS-CoV-2 delta derivative and reinfection with Omicron variant in a fully vaccinated health care professional. J Infect. 2022;85:e15. 10.1016/j.jinf.2022.04.023. 35447232 10.1016/j.jinf.2022.04.023 PMC9014656

[50] Kafwanka RC, Chipimo M, Incidence. Risk Factors, and outcomes of severe breakthrough COVID-19 infections among hospitalized patients in Africa. Int Res Med Health Sci. 2024;7:7–21. 10.36437/irmhs.2024.7.2.B.

[51] Huai Luo C, Paul Morris C, Sachithanandham J, Amadi A, Gaston DC, Li M, et al. Infection with the severe acute respiratory syndrome coronavirus 2 (SARS-CoV-2) delta variant is associated with higher recovery of infectious virus compared to the alpha variant in both unvaccinated and vaccinated individuals. Clin Infect Dis. 2021;75:e715–25. 10.1093/cid/ciab986. 10.1093/cid/ciab986 PMC8903351 34922338

[52] Tada T, Zhou H, Samanovic MI, Dcosta BM, Cornelius A, Herati RS, et al. Neutralization of SARS-CoV-2 variants by mRNA and adenoviral vector vaccine-elicited antibodies. Front Immunol. 2022. 10.3389/fimmu.2022.797589. 35350781 10.3389/fimmu.2022.797589 PMC8957851

[53] Stamatatos L, Czartoski J, Wan Y-H, Homad LJ, Rubin V, Glantz H, et al. mRNA vaccination boosts cross-variant neutralizing antibodies elicited by SARS-CoV-2 infection. Science. 2021;372:1413–8. 10.1126/science.abg9175. 33766944 10.1126/science.abg9175 PMC8139425

[54] Duerr R, Dimartino D, Marier C, Zappile P, Wang G, Lighter J, et al. Dominance of alpha and iota variants in SARS-CoV-2 vaccine breakthrough infections in New York City. J Clin Invest. 2021. 10.1172/JCI152702. 34375308 10.1172/JCI152702 PMC8439605

[55] Dingemans J, van der Veer BMJW, Gorgels KMF, Hackert V, den Heijer CDJ, Hoebe CJPA, et al. Investigating SARS-CoV-2 breakthrough infections per variant and vaccine type. Front Microbiol. 2022. 10.3389/fmicb.2022.1027271. 36504818 10.3389/fmicb.2022.1027271 PMC9729533

[56] Asare AF, Sabblah GT, Buabeng RO, Alhassan Y, Asamoa-Amoakohene A, Amponsa-Achiano K, et al. Adverse events following COVID-19 vaccination: a comprehensive analysis of spontaneous reporting data in Ghana. PLOS Glob Public Health. 2024;4:e0003770. 10.1371/journal.pgph.0003770. 39331603 10.1371/journal.pgph.0003770 PMC11432875

[57] Bowen JE, Addetia A, Dang HV, Stewart C, Brown JT, Sharkey WK, et al. Omicron spike function and neutralizing activity elicited by a comprehensive panel of vaccines. Science. 2022;377:890–4. 10.1126/science.abq0203. 35857529 10.1126/science.abq0203 PMC9348749

[58] Otto SP, Day T, Arino J, Colijn C, Dushoff J, Li M, et al. The origins and potential future of SARS-CoV-2 variants of concern in the evolving COVID-19 pandemic. Curr Biol. 2021;31:R918–29. 10.1016/j.cub.2021.06.049. 34314723 10.1016/j.cub.2021.06.049 PMC8220957

[59] Xie J, Ye F, Deng X, Tang Y, Liang J-Y, Huang X, et al. Circular RNA: a promising new star of vaccine. J Transl Intern Med. 2023;11:372–81. 10.2478/jtim-2023-0122. 10.2478/jtim-2023-0122 PMC10732498 38130633

[60] Bongiovanni M, Spada E, De Angelis C, Liuzzi G, Giuliani G. SARS-CoV-2 re-infection, vaccination and neutralizing antibodies. J Infect. 2022;84:e120–1. 10.1016/j.jinf.2022.02.033. 35245582 10.1016/j.jinf.2022.02.033 PMC8886678

[61] Guo M, Shang S, Li M, Cai G, Li P, Chen X, et al. Understanding autoimmune response after SARS-CoV-2 infection and the pathogenesis/mechanisms of long COVID. Med Rev 2021. 2024;4:367–83. 10.1515/mr-2024-0013. 39444797 10.1515/mr-2024-0013 PMC11495526

[62] Ewing AG, Salamon S, Pretorius E, Joffe D, Fox G, Bilodeau S, et al. Review of organ damage from COVID and long COVID: a disease with a spectrum of pathology. Med Rev 2021. 2025;5:66–75. 10.1515/mr-2024-0030. 39974559 10.1515/mr-2024-0030 PMC11834749

