# Correction: Cycle threshold values and SARS-CoV-2 variant associations with breakthrough infections: a retrospective study in Accra, Ghana

**DOI:** 10.1186/s12879-025-11965-5

**Published:** 2025-10-23

**Authors:** Frank Twum Aboagye, Lawrence Annison, Ebenezer Krampah Aidoo, Maame Ekua Acquah, Yvonne Aryeetey Ashong, Betty Bandoh Oppong, Lawrencia Osae-Nyarko, Isaac Owusu-Frimpong, Henry Kwadwo Hackman, Sharon Annison, Queenstar Dedei Quarshie, Abena Konadu Owusu-Senyah Enninful, Naa Adjeley Kuma, Bill Clinton Egyam, Mike Y. Osei-Atweneboana

**Affiliations:** 1 Department of Medical Laboratory Technology, Faculty of Applied Sciences, Accra Technical University, Accra, Ghana; 2 Biomedical and Public Health Research Unit, Council for Scientific and Industrial Research – Water Research Institute, Accra, Ghana; 3 West African Centre for Cell Biology of Infectious Pathogens, College of Basic and Applied Sciences, University of Ghana, Legon, Accra, Ghana; 4 Department of Parasitology, College of Medical Sciences, Noguchi Memorial Institute of Medical Research, University of Ghana, Legon, Accra, Ghana; 5 Department of Molecular Microbiology and Immunology, Bloomberg School of Public Health, Johns Hopkins University, Baltimore, MD USA; 6 Department of Epidemiology and Disease Control, School of Public Health, University of Ghana, Legon, Accra, Ghana; 7 Department of Molecular Biology, MDS Lancet Laboratories Ghana Limited, East Legon, Accra, Ghana; 8 Council for Scientific and Industrial Research – College of Science and Technology, Accra, Ghana

**Correction: BMC Infectious Diseases (2025) 25:1269**.

10.1186/s12879-025-11732-6.

Following publication of the original article [1], we were notified that the updated version for Fig. 2 submitted during proofing was not used in Production.

Originally published Fig. 2:

**Figure 1:**
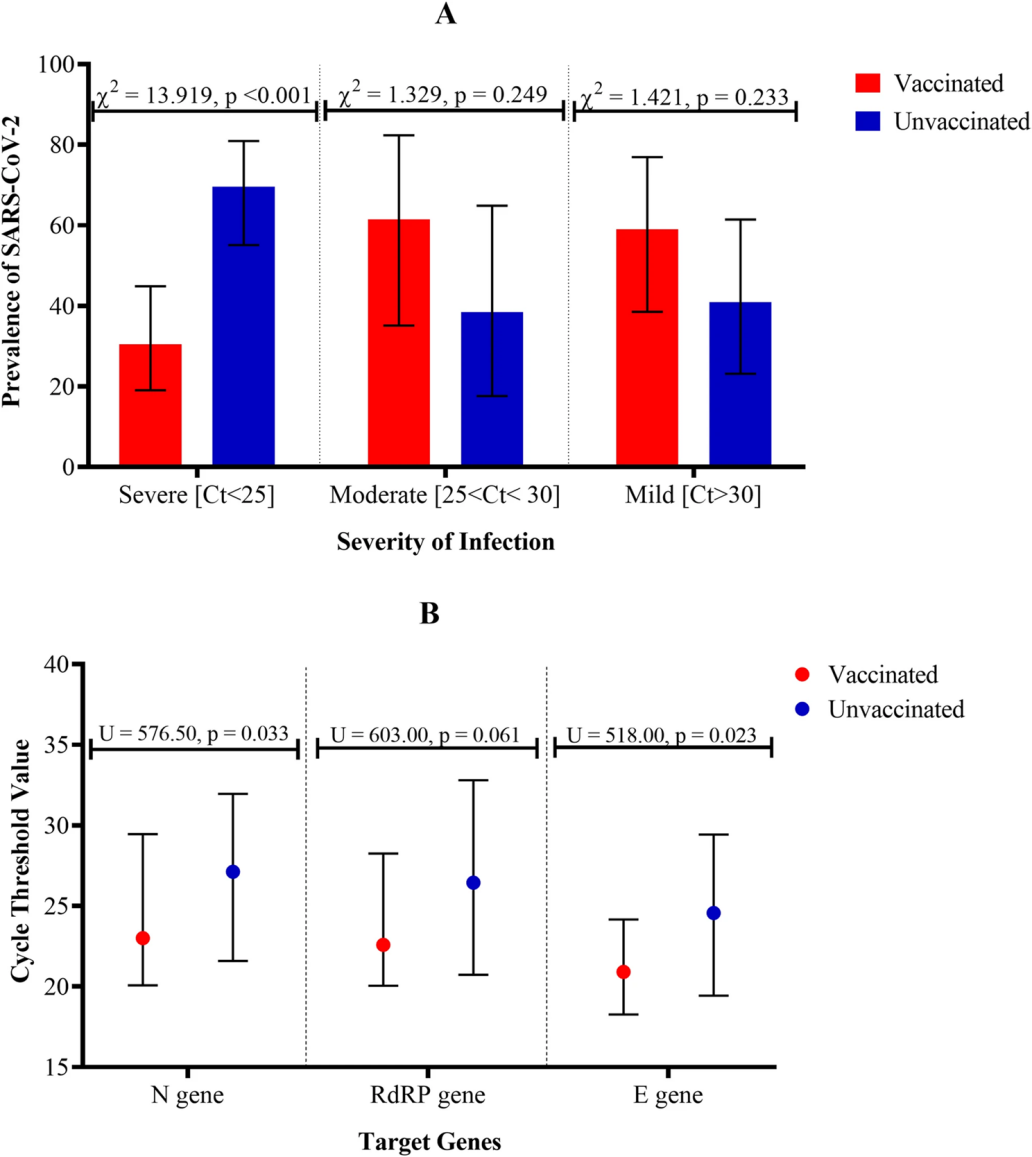
Fig. 2

Corrected Fig. 2:

**Figure 2:**
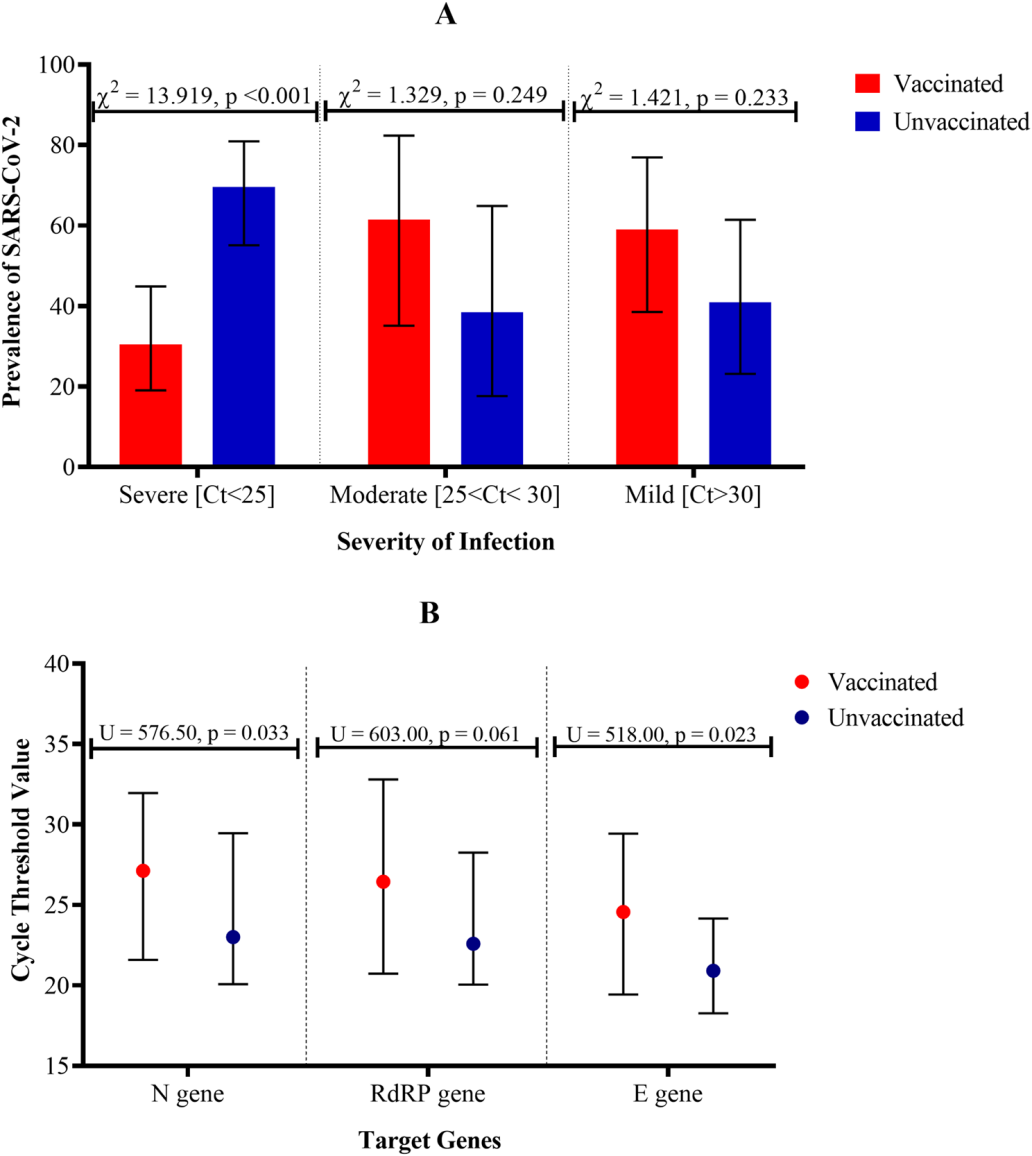
Fig. 2

The original article has been corrected.
