# Supplementary material for: Cycle threshold values and SARS-CoV-2 variant associations with breakthrough infections: a retrospective study in Accra, Ghana
Source: BMC Infect Dis. 2025 Oct 10;25:1269. doi: 10.1186/s12879-025-11732-6 (PMC12513011; doi:10.1186/s12879-025-11732-6)
Supplement: Supplementary file 1 — Supplementary Material 1 [file 12879_2025_11732_MOESM1_ESM.docx]

**Table S1: Vaccination Characteristics of the Study Population**

|  | **Number of Participants** | **Percentage** |
| --- | --- | --- |
| **Vaccine Brand** |  |  |
| Sputnik V | 7 | 20.00 |
| Pfizer-BioNTech | 10 | 28.57 |
| Johson & Johnson | 12 | 34.29 |
| AstraZeneca | 6 | 17.14 |
| **Interval (days) from last dose to sampling** |  |  |
| Median Days | 111.3 [IQR: 49 - 165] |  |
| 15 – 30 | 4 | 11.40 |
| 31 – 90 | 10 | 28.60 |
| > 90 | 21 | 60.00 |
