# Supplementary material for: Cycle threshold values and SARS-CoV-2 variant associations with breakthrough infections: a retrospective study in Accra, Ghana
Source: BMC Infect Dis. 2025 Oct 10;25:1269. doi: 10.1186/s12879-025-11732-6 (PMC12513011; doi:10.1186/s12879-025-11732-6)
Supplement: Supplementary file 3 — Supplementary Material 3 [file 12879_2025_11732_MOESM3_ESM.docx]

**Table S3: Vaccination (brand) stratified by age and Ct-values**

| **Variables** | **Vaccine Brand (n[%])** | | | | **Total** |
| --- | --- | --- | --- | --- | --- |
|  | **Sputnik V**  **[n = 7]** | **Pfizer-BioNTech**  **[n = 10]** | **Johson & Johnson**  **[n = 12]** | **AstraZeneca**  **[n = 6]** |  |
| **Age (years)** |  |  |  |  |  |
| ≤ 20 | 1 [14.29] | 1 [10.00] | 1 [8.33] | 0 [0.00] | 3 [8.57] |
| 21 - 40 | 5 [71.43] | 4 [40.00] | 5 [41.67] | 2 [33.33] | 16 [45.71] |
| 41 - 60 | 0 [0.00] | 2 [20.00] | 5 [41.67] | 3 [50.00] | 10 [28.57] |
| > 60 | 1 [14.29] | 3 [30.00] | 1 [8.33] | 1 [16.67] | 6 [17.14] |
| **Ct-values** |  |  |  |  |  |
| Ct < 25 | 2 [28.57] | 4 [40.00] | 5 [41.67] | 3 [50.00] | 14 [40.00] |
| 25 < Ct < 30 | 1 [14.29] | 3 [30.00] | 2 [16.67] | 2 [33.33] | 8 [22.86] |
| Ct > 30 | 4 [57.14] | 3 [30.00] | 5 [41.67] | 1 [16.67] | 13 [37.14] |

Values reported are number of participants (n) and their corresponding proportion of the sub-group (%)
